# Three new genera of acidocerine water scavenger beetles from tropical South America (Coleoptera, Hydrophilidae, Acidocerinae)

**DOI:** 10.3897/zookeys.768.24423

**Published:** 2018-06-19

**Authors:** Jennifer C. Girón, Andrew Edward Z. Short

**Affiliations:** 1 Department of Ecology & Evolutionary Biology, and Division of Entomology, Biodiversity Institute, University of Kansas, Lawrence, KS 66045, USA

**Keywords:** aquatic beetles, new species, taxonomy, seepage habitat

## Abstract

Recent collecting efforts in the Neotropics have led to the discovery of numerous new species and lineages of aquatic beetles. Here, three new genera are described to accommodate fifteen new species of water scavenger beetles of the subfamily Acidocerinae from northern South America: *Crucisternum* **gen. n.** for *C. escalera* **sp. n.** (Venezuela), *C. ouboteri* **sp. n.** (Guyana, French Guiana, Suriname, Venezuela), *C. queneyi* **sp. n.** (French Guiana), *C. sinuatus* **sp. n.** (Brazil), *C. toboganensis* **sp. n.** (Venezuela), *C. vanessae* **sp. n.** (Suriname), and *C. xingu* **sp. n.** (Brazil); *Katasophistes* **gen. n**. for *K. charynae* **sp. n.** (Peru), *K. cuzco* **sp. n.** (Peru), *K. merida* **sp. n.** (Venezuela) and *K. superficialis* **sp. n.** (Ecuador); and *Nanosaphes* **gen. n.** for *N. castaneus* **sp. n.** (Brazil), *N. hesperus* **sp. n.** (Suriname), *N. punctatus* **sp. n.** (Guyana), and *N. tricolor* **sp. n.** (Guyana, Suriname). It was also found that the monotypic Neotropical endemic genus *Dieroxenus* Spangler, 1979, **syn. n.** is congeneric with *Chasmogenus* Sharp, 1882 resulting in the single new combination *Chasmogenus cremnobates* (Spangler, 1979), **comb. n.**. *Katasophistes merida* **sp. n.** is known exclusively from seepage habitats, while the remaining taxa described herein are primarily associated with the margins of densely forested streams. Diagnoses, illustrations, distribution maps, and habitat summaries are provided for all new genera and species. A key to the genera of Acidocerinae of the New World is provided.

## Abstract (es)

Recientes esfuerzos de colecta en el neotrópico han llevado al descubrimiento de numerosas especies y linajes nuevos de escarabajos acuáticos. Aquí se describen tres géneros nuevos, para acomodar quince especies nuevas de escarabajos acuáticos de la subfamilia Acidocerinae, del norte de Suramérica: *Crucisternum* **gen. n.** para *C. escalera* **sp. n.** (Venezuela), *C. ouboteri* **sp. n.** (Guyana, Guyana Francesa, Surinam, Venezuela), *C. queneyi* **sp. n.** (Guyana Francesa), *C. sinuatus* **sp. n.** (Brasil), *C. toboganensis* **sp. n.** (Venezuela), *C. vanessae* **sp. n.** (Surinam), y *C. xingu* **sp. n.** (Brasil); *Katasophistes* **gen. n**. para *K. charynae* **sp. n.** (Perú), *K. cuzco* **sp. n.** (Perú), *K. merida* **sp. n.** (Venezuela) y *K. superficialis* **sp. n.** (Ecuador); y *Nanosaphes* **gen. n.** para *N. castaneus* **sp. n.** (Brazil), *N. hesperus* **sp. n.** (Surinam), *N. punctatus* **sp. n.** (Guyana), y *N. tricolor* **sp. n.** (Guyana, Surinam). También se encontró que el género *Dieroxenus* Spangler, 1979, **syn. n.**, el cual es monotípico, neotropical y endémico, es congenérico con *Chasmogenus* Sharp, 1882, lo que resulta en la nueva combinación *Chasmogenus cremnobates* (Spangler, 1979), **comb. n.**. *Katasophistes merida* **sp. n.** se conoce exclusivamente de hábitats higropétricos, mientras que los taxa restantes que se describen aquí, están primordialmente asociados con los márgenes de corrientes densamente forestadas. Diagnosis, ilustraciones, mapas de distribución y un resumen de los hábitats, son provistos para todos los géneros y especies nuevas. Se provee además una clave para la identificación de géneros de Acidocerinae del nuevo mundo.

## Introduction

Recent fieldwork in northern South America has significantly expanded our knowledge of water beetle diversity, not only in terms of the number of species, but also in illuminating the extraordinary diversity of habitats that they occupy (Short 2018). In particular, many new apparent forms of the water scavenger beetle subfamily Acidocerinae have been identified from a range of habitats from seepages to forest streams to savanna ponds. The Acidocerinae presently contains 14 genera and about 300 species (Short and Fikáček 2011, 2013). Although they are distributed worldwide, most acidocerine species occur in tropical regions. Four new Neotropical genera have already been described in the last twenty years: *Quadriops* Hansen, 1999, *Globulosis* García, 2001, *Tobochares* Short & García, 2007, and *Radicitus* Short & García, 2014. An ongoing review of the phylogeny and generic concepts within the subfamily has led to the discovery of yet more taxa that cannot be accommodated within existing genera. Here we describe three of these new genera to accommodate fifteen new species from tropical South America. In the course of this work we also discovered a new generic junior synonym, which we formally designate as well.

## Materials and methods

Depositories of examined material:

**CBDG** Center for Biological Diversity, University of Guyana, Georgetown

**INPA** Instituto Nacional de Pesquisas da Amazônia, Manaus, Brazil)

**MALUZ** Museo de Artrópodos de la Universidad del Zulia, Maracaibo, Venezuela (J. Camacho, M. García)

**MIZA** Museo del Instituto de Zoología Agrícola, Maracay, Venezuela

**MNHN** Muséum National d’Histoire Naturelle, Paris, France

**NHMUK** The Natural History Museum, London, United Kingdom (formerly British Museum (Natural History BMNH; M. Barclay, C. Taylor)

**NZCS** National Zoological Collection of Suriname, Paramaribo (P. Ouboter, V. Kadosoe)

**PQPC** Personal collection of Pierre Queney, Paris, France.

**PUCE** Pontificia Universidad Católica del Ecuador, Quito, Ecuador (C. Kiel)

**SEMC** Snow Entomological Collection, University of Kansas, Lawrence, KS (A. Short)

**USNM** U.S. National Museum of Natural History, Smithsonian Institution, Washington, DC (C. Micheli).

Over 650 specimens were examined. Specimen preparation and examination methods are identical to those given in Girón and Short (2017). Dissected male genitalias were packed in microvials with glycerin and pinned under the specimens, except for the species of *Nanosaphes*, for which each minute genitalia was glued onto the specimen point using alcohol soluble translucent glue.

Descriptive sequence and morphological terminology largely follows Hansen (1991) except for the use of meso- and metaventrite instead of meso- and metasternum, and abdominal ventrites instead of abdominal sternites (see Lawrence and Ślipiński 2013). Terms for the ventral surface of head follows Komarek (2004). Terminology for the metafurca follows Velázquez de Castro (1998).

Descriptions of genera and species are organized in alphabetical order, whereas in the habitus figures species are grouped by similarity for ease of comparison.

All specimen data which can be searched by species and/or collecting event are available online through the Collection Resources for Aquatic Coleoptera (CReAC) portal at http://creac.kubiodiversityinstitute.org/collections/.

## Results

### List of species and their provenance

***Crucisternum* gen. n.**

1. *Crucisternum escalera* **sp. n.** Venezuela (Bolívar)

2. *Crucisternum ouboteri* **sp. n.** French Guiana, Guyana, Suriname, Venezuela (Amazonas)

3. *Crucisternum queneyi* **sp. n.** French Guiana

4. *Crucisternum sinuatus* **sp. n.** Brazil (Minas Gerais, Pará)

5. *Crucisternum toboganensis* **sp. n.** Venezuela (Amazonas)

6. *Crucisternum vanessae* **sp. n.** Suriname

7. *Crucisternum xingu* **sp. n.** Brazil (Pará)

***Katasophistes* gen. n.**

8. *Katasophistes charynae* **sp. n.** Peru (Madre de Dios)

9. *Katasophistes cuzco* **sp. n.** Peru (Cuzco)

10. *Katasophistes merida* **sp. n.** Venezuela (Mérida)

11. *Katasophistes superficialis* **sp. n.** Ecuador (Pastaza)

***Nanosaphes* gen. n.**

12. *Nanosaphes castaneus* **sp. n.** Brazil (Pará)

13. *Nanosaphes hesperus* **sp. n.** Guyana, Suriname

14. *Nanosaphes punctatus* **sp. n.** Guyana, Suriname

15. *Nanosaphes tricolor* **sp. n.** Guyana, Suriname

## Taxonomy

### Crucisternum gen. n.

Taxon classificationAnimaliaColeopteraHydrophilidae

http://zoobank.org/9235AE60-8431-4ECB-9C93-F13D4C663F9F

Figs 1 , 2 , 3 , 4 , 5 , 6 , 7 , 8

#### Type species.

*Crucisternum ouboteri*.

#### Differential diagnosis.

**Figure 1.**
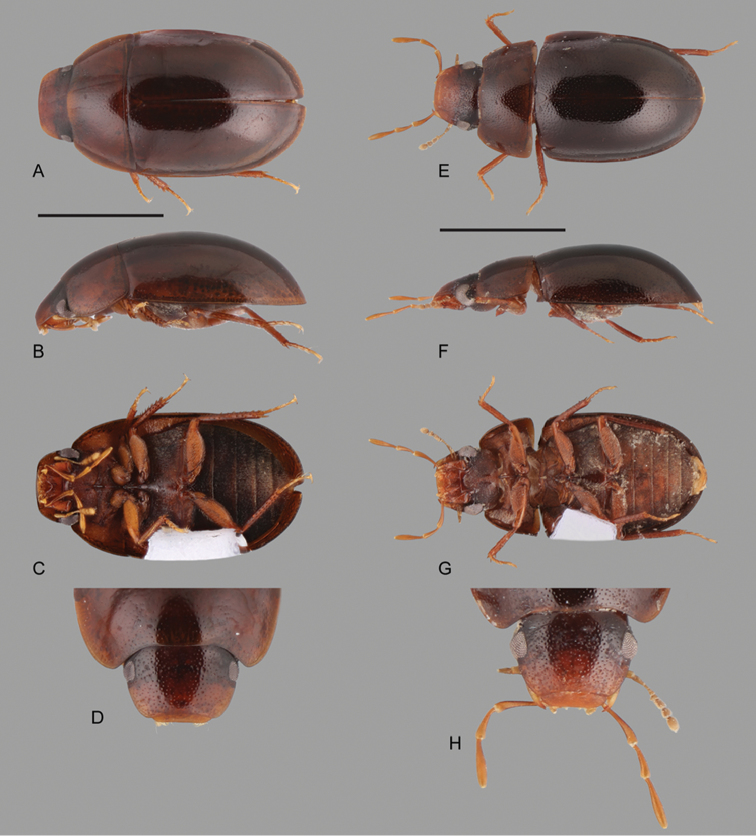
Habitus of *Crucisternum* spp.: **A–D** *Crucisternum escalera*: **A** dorsal view **B** lateral view **C** ventral view **D** head **E–H** *Crucisternum queneyi*: **E** dorsal view **F** lateral view **G** ventral view **H** head. Scale bars: 1 mm.

**Figure 2.**
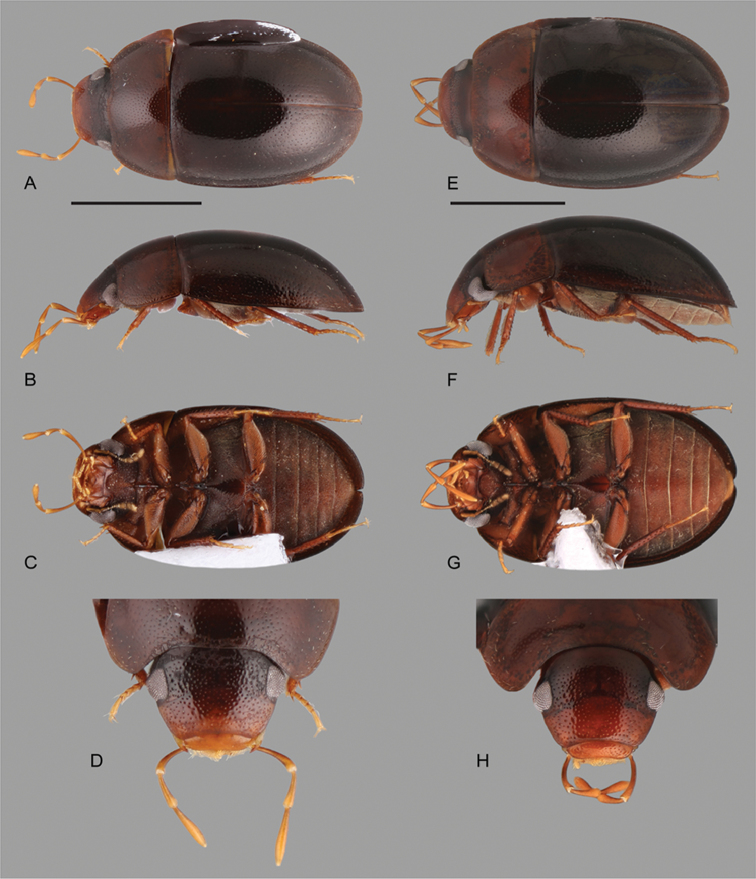
Habitus of *Crucisternum* spp.: **A–D** *Crucisternum ouboteri*: **A** dorsal view, **B** lateral view **C** ventral view, **D** head **E–H** *Crucisternum vanessae*: **E** dorsal view **F** lateral view **G** ventral view **H** head. Scale bars: 1 mm.

**Figure 3.**
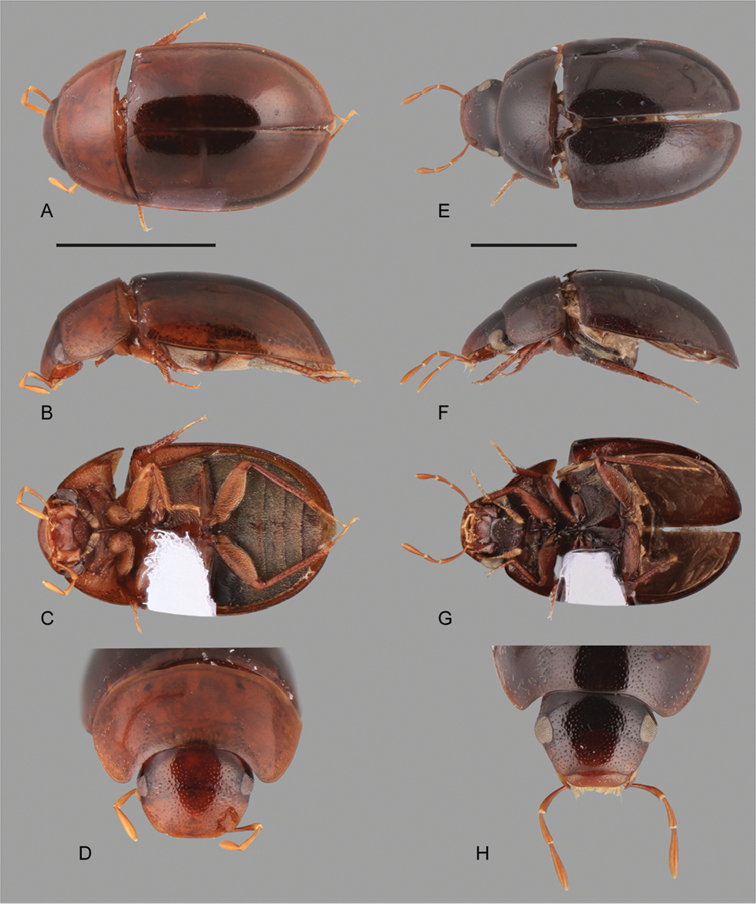
Habitus of *Crucisternum* spp.: **A–D** *Crucisternum sinuatus*: **A** dorsal view **B** lateral view **C** ventral view **D** head **E–H** *Crucisternum xingu*: **E** dorsal view **F** lateral view **G** ventral view **H** head. Scale bars: 1 mm.

**Figure 4.**
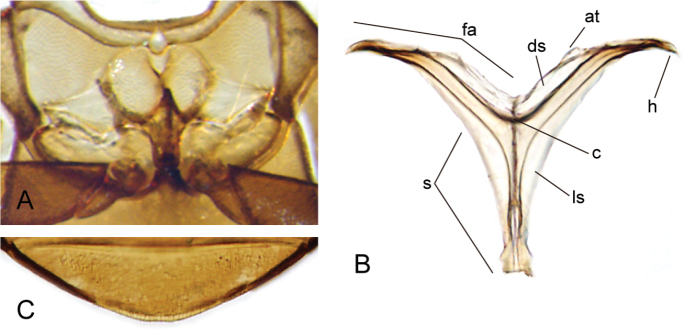
Thorax and abdomen of *Crucisternum ouboteri*: **A** ventral view of mesoventrite (white arrow pointing longitudinal carina and transverse ridge); **B** posterior view of metafurca: (at) anterior tendon, (c) crux, (ds) dorsal sheath, (fa) furcal arm, (h) hemiductus, (ls) lateral sheath, (s) stalk; **C** fifth abdominal ventrite.

**Figure 5.**
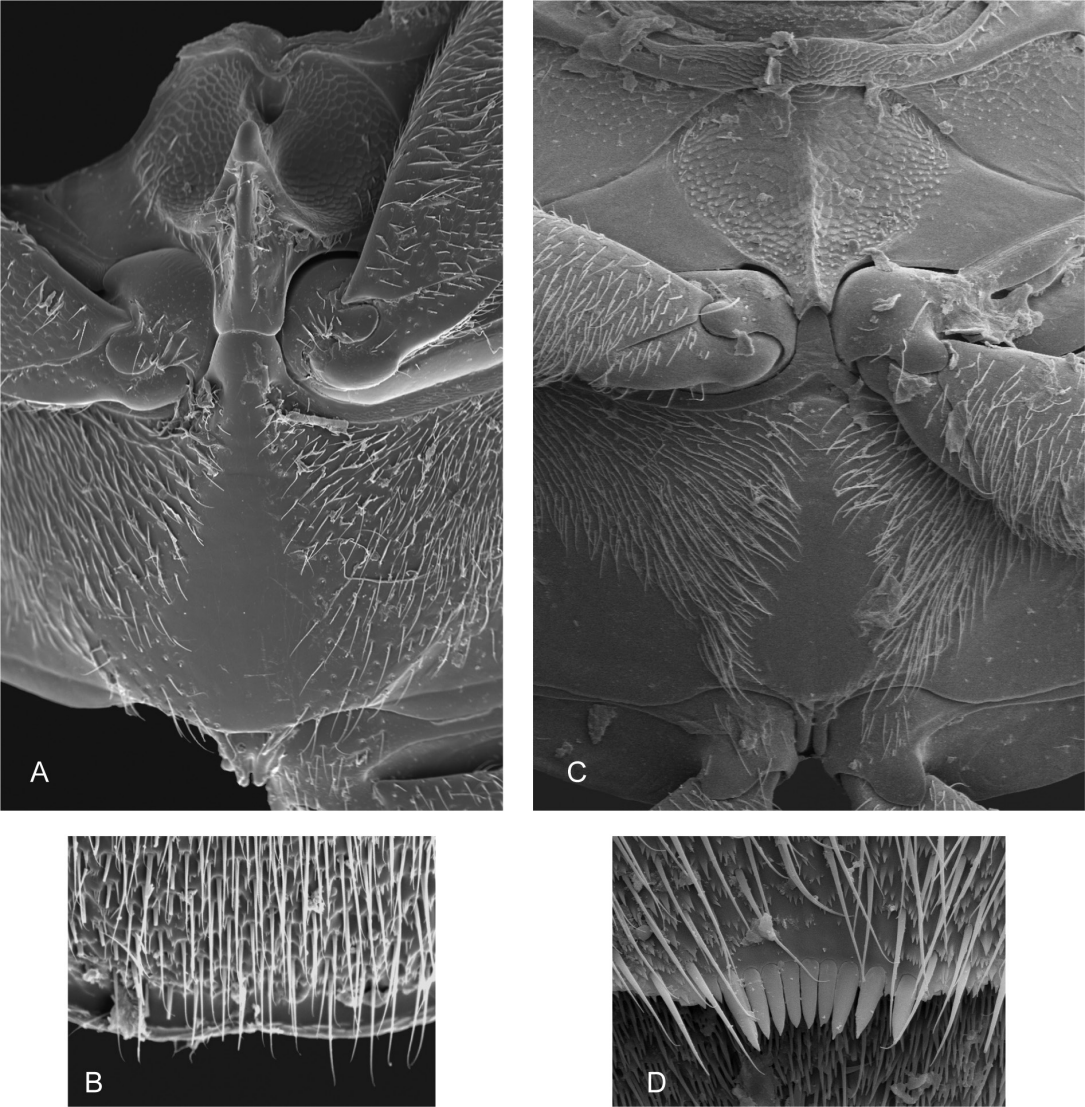
SEM images of thorax and abdomen: **A–B** *Crucisternum ouboteri*: **A** meso- and metaventrite **B** apex of fifth abdominal ventrite; **C–D** *Nanosaphes tricolor* **C** meso- and metaventrite **D** apex of fifth abdominal ventrite.

Small beetles, total body length 2.0–2.5 mm, width 1.1–1.4 mm. Color orange brown to dark brown. Body shape elongated oval in dorsal view; moderately convex in lateral view (see Figs 1–3). Antennae with nine antennomeres (e.g., Fig. 2C). Maxillary palps curved inward, moderately long (e.g., Fig. 2D). Elytra without sutural striae; serial punctures, ground punctures and systematic punctures similar in size and degree of impression, either shallow or rather sharply marked; all punctures seemingly arranged in rows; outer margins of elytra slightly flared. Prosternum with a well-developed median longitudinal carina (e.g., Fig. 3B). Posterior elevation of mesoventrite with a strongly produced, anteriorly pointed transverse ridge, longitudinally carinate (Figs 4A, 5A). Posterior femora glabrous at most along apical fifth. Fifth abdominal ventrite apically rounded, truncate or slightly emarginate, without stout setae (e.g., Figs 1G, 4C, 5B).

Although *Crucisternum* is generally unremarkable dorsally from other small-bodied acidocerines, several sternal features easily separate the genus from all others. Within the Acidocerinae, the strongly-developed prosternal carina found in the genus is extremely rare, occurring only in the Afrotropical genus *Acidocerus*. Additionally, the strongly produced postero-medial projection of the mesoventrite, formed by the fusion of both transverse and longitudinal ridges (Figs 4A, 5A), is unique in the subfamily. It is most likely to be confused in samples as a small *Chasmogenus*, but can also easily be distinguished from that genus by the lack of a sutural stria.

#### Description.

**Figure 6.**
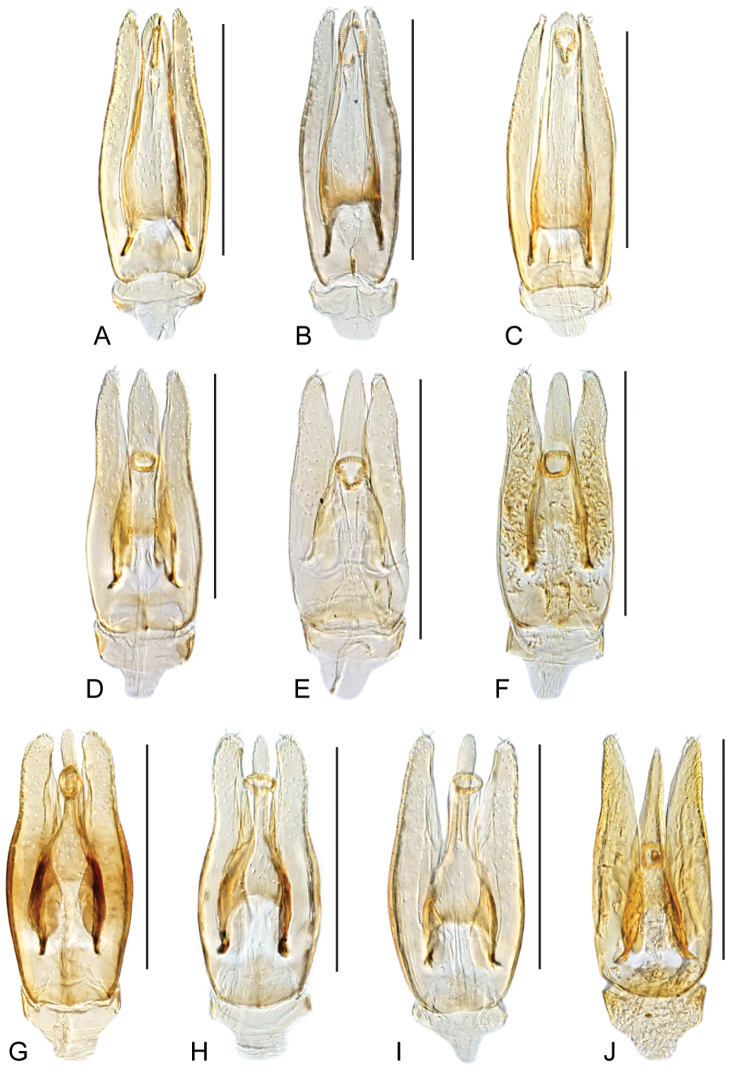
Aedeagus of *Crucisternum* spp.: **A–B** *Crucisternum ouboteri*: **A** Guyana **B** Brownsberg **C** *Crucisternum toboganensis* **D** *Crucisternum vanessae* **E–F** *Crucisternum xingu*: **E** colln#6 **F** colln#23 **G–H** *Cuscisternum sinuatus*: **G** colln#23 **H** BR18-0302-04A **I** *Crucisternum escalera* **J** *Crucisternum queneyi*. Scale bars: 0.25 mm.

Body elongate oval, moderately convex, orange brown to dark brown in color, sometimes slightly paler on ventral surface and appendages. **Head.** Frons and clypeus (e.g., Fig. 2D) with moderately marked ground punctures, irregularly and rather densely distributed over the surface, accompanied by scattered seta-bearing systematic punctures, particularly noticeable on anterior area of frons; surface between punctures smooth and shiny; anterior corners of clypeus roundly angulated; anterior margin of clypeus widely roundly emarginate. Eyes subquadrate in dorsal view. Labrum wide, fully exposed, nearly half as long, and collinear to perpendicular to clypeus (e.g., Fig. 2H); dorsal surface slightly convex, with scattered fine punctures; anterior margin roundly bent inwards, mesally emarginate and with tiny denticles along emargination; lateral and anterior margins fringed by rather long setae. Temporae densely covered by very short and fine setae (hydrofuge pubescence). Mentum parallel sided, with lateral oblique longitudinal ridges; anterior margin with wide, deep, concave median impression marked by a u- or bell-shaped transverse carina. Submentum sunken and pubescent at base, glabrous, shiny and ascending at apex; ocular ridge well-developed (e.g., Fig. 1G). Maxilla (see Fig. 2C) with ventral surface of cardo and stipes smooth and shiny, with scattered and shallow punctures; outer dorsal margin of palpifer with a row of stiff, decumbent, curved, spiniform setae; limit between cardo and stipes oblique; maxillary palps brown to orange, longer than antennae, with palpomere I usually darker at base, gradually paler towards apex; apex of palpomere 3 bearing sensilla. Mandibles with apex bifid (examined in *C. ouboteri* and *C. sinuatus*). Labial palps yellowish to brown, usually shorter than mentum, at most nearly as long, dorsoventrally flattened; palpomere 2 with outer margin convex apicad of midpoint, sometimes with one (preapical) or two (one median, one preapical) setae on outer surface; palpomere 3 digitiform, with a long subapical seta on outer corner. Antennae (see Fig. 2C) with 9 antennomeres, either similar or paler than general coloration of head; antennomere 1 anteriorly projected near base, at most reaching midpoint of ventral surface of eye (not reaching cardo-stipes joint), nearly 1.7–2.5-times longer than antennomere 2; antennomere 2 nearly as long as antennomeres 3–5 combined; antennomere 6 forming a well differentiated, symmetric cupule; antennomeres 7–9 slightly flattened, forming a loosely articulated, pubescent club (antennomere 8 shortest, 9 longest); apex of antennomere 9 with longer setae than general pubescence of club. **Thorax.** Pronotum widest at base, narrowed anteriorly, surface evenly convex; ground punctation moderate, uniformly dense, with surface between punctures smooth and shiny; seta bearing systematic punctures forming paired anterolateral semicircles. Scutellar shield of moderate size, triangular, nearly as long as wide, with punctation as in pronotum. Prosternum (e.g., Fig. 2C) nearly as long as 2/3 the length of a procoxa, with a strong median longitudinal carina; anterior margin of prosternum mesally projected as a wide triangle; surface of prosternum usually convex and with scarce crenulations, with scattered, rather long, fine setae; intercoxal process projected from posterior margin of procoxal cavities, triangularly shaped in outline, mesally longitudinally carinate. Mesoventrite (Figs 4A, 5A) not fused to mesepisterna, with anterior margin nearly 0.3-times as wide as anterior margin of mesepisternum; anterior rib of mesoventrite bearing a medial teardrop-shaped, pearlescent macula; posterior elevation of mesoventrite with a strongly produced, anteriorly pointed transverse ridge, longitudinally carinate, bearing scarce, rather long setae; surface of mesoventrite reticulated for the most part, with an anteromedial depression, and posterolateral smooth and glabrous areas; mesepisternum obliquely widely concave, with reticulated surface; mesepimeron trapezoid, with reticulate and pubescent surface. Mesofurca (examined in *C. ouboteri*) with short arms, 0.8-times length of mesocoxae; apical half of arms free, irregularly explanate at apex. Metaventrite mesally elevated, with elevation smooth and glabrous (Fig. 5A), rather narrow anteriorly, wide and flat posteriorly; surface of metaventrite densely pubescent, except for median and postero-lateral glabrous areas. Metepisterna approximately 3-times longer than wide, narrowing only at posterior end. Metepimeron triangular and acute posteriorly. Metafurca (examined in *C. ouboteri*, Fig. 4B) 1.45-times wider than long, with furcal arms (fa) slightly longer than stalk (s); stalk triangular (wider near the crux (c), gradually narrowing ventrally), with paired longitudinal keels extending along basal third of posterior face, fusing together towards crux, with a well-developed median keel on anterior face extending to anterior margin of dorsal sheets (ds); outer margins of stalk diverging from basal third towards distal third of furcal arms; furcal arms somewhat trapezoid, with apex (hemiductus (h)) only slightly explanate, with apex pointing obliquely; anterior tendons (at) inserted at mid length of dorsal edge of furcal arms; dorsal sheaths well-developed, wider than widest point of lateral sheaths (ls). **Elytra.** Surface even (without elevations or depressions), without sutural striae; serial punctures, ground punctures and systematic punctures similar in size and degree of impression, either shallow or rather sharply marked; all punctures seemingly arranged in rows; serial punctures not impressed into striae; seta bearing systematic punctures rather scarce; elytral margins slightly flared. Epipleura well-developed, surface slightly oblique, with sparse setae and irregular sculpture, anteriorly wide, gradually narrowing posteriorly, extending up to line of posterior margin of first abdominal ventrite; inner margin of epipleura with small indentation articulating anterior outer corner of metepisternum; pseudepipleura well-developed, perpendicularly positioned, nearly half as wide as anterior portion of epipleura, extending along entire outer margin of elytra. Hind wings well-developed. **Legs.** All femora with dense pubescence, except on at most apical fifth, in which surface is glabrous, shiny and slightly reticulated; all femora antero-posteriorly flattened; metafemora with rather weak tibial grooves. Tibiae slender, rather cylindrical; well-developed spines along pro- and mesotibiae, smaller and sparser in metatibiae; protibiae with a median longitudinal row of spathulate setae along anterior surface. All tarsi with five tarsomeres, bearing long apical hair-like setae on dorsal face, and two lateral rows of spines and/or hair-like spines on ventral face of tarsomeres 2–4; tarsomeres 1–4 similar in size and shape; tarsomere 5 approximately as long as tarsomeres 3–4 combined, without spines on ventral face; claws rather large, curved; empodium well-developed, bearing a pair of long, curved apical setae. **Abdomen.** Abdomen with five ventrites, medially convex; all ventrites with uniform, dense, fine pubescence; posterior margin of fifth ventrite uniformly rounded, truncate or slightly emarginate, without thick, flat spine-like setae (Figs 4C, 5B). Aedeagus (Fig. 6) with basal piece between 0.2 and 0.25-times the length of parameres; apical half of median lobe wider to narrower than a paramere; median lobe with well-developed lateral basal apodemes, and acute to narrowly rounded apex; parameres nearly as long as median lobe, with outer margins usually sinuate, usually with setae at apex; gonopore situated distad of mid length of median lobe.

#### Larvae.

The immature stages are unknown.

#### Etymology.

Named from the Latin *crucis*, meaning cross, combined with the word sternum, in reference to the conspicuous cruciform elevation of the mesoventrite in species of the genus. To be treated as masculine.

#### Distribution.

**Figure 7.**
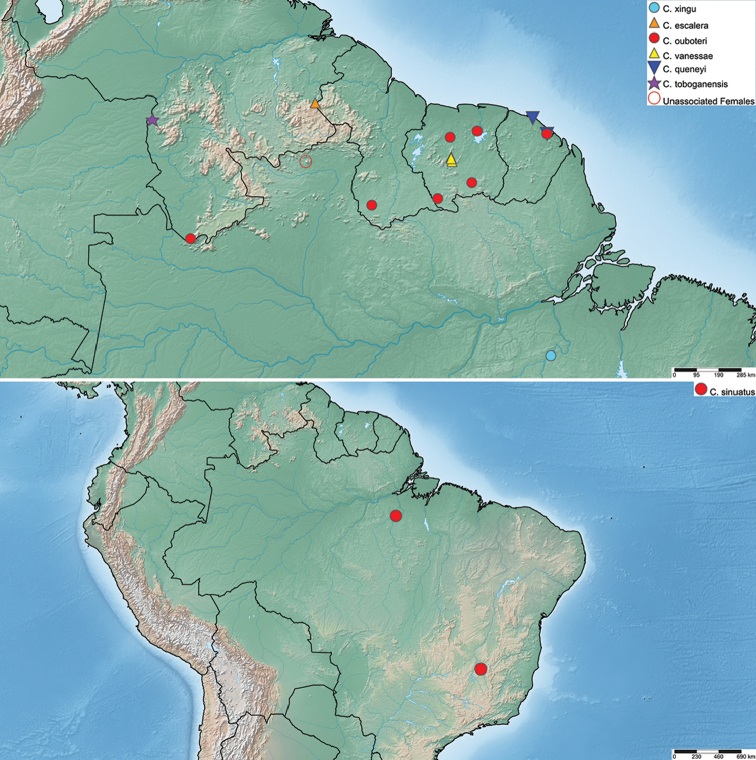
Distribution of *Crucisternum* species.

Brazil, Guyana, French Guiana, Suriname, Venezuela. See Fig. 7.

#### Biology.

Without exception, all species of the genus are associated with forested streams, usually along margins that contain ample detritus. A single specimen of *C. ouboteri* was collected at a black light trap.

#### Characters of taxonomic importance for *Crucisternum*.

The external morphology of *Crucisternum* is highly homogeneous across species, with characters of the aedeagus being the most reliable for species identification, in particular for those with sharply marked elytral punctures. Unassociated female specimens may not be able to be authoritatively identified in some cases.

***Punctation*.** Two groups of species can be recognized by the degree of impression of the elytral ground punctures: *Crucisternum toboganensis*, *C. ouboteri*, *C. queneyi*, and *C. vanessae* exhibit rather sharply marked punctures, whereas the ground punctures of *C. escalera*, *C. sinuatus*, and *C. xingu* are more shallowly impressed (compare Fig. 1A to Fig. 1E).

***Coloration*.** The dorsal coloration of the body can be either uniform as in *C. escalera* (Fig. 1A), and *C. xingu*, or the elytra can be darker than the head and pronotum, as in *C. ouboteri* (Fig. 2A), *C. queneyi*, and *C. vanessae*. Specimens of *C. sinuatus* collected in 1986 in northern Brazil were preserved in 70% alcohol until the time of this revision, and are darker in coloration, whereas the freshly collected specimens from Minas Gerais, are paler (orange brown). It is possible that the alcohol affected the coloration of the specimens over time causing them to darken. General coloration (e.g., light vs. dark brown) should not be used exclusively as diagnostic.

***Aedeagus*.** The basal piece is strongly reduced in *Crucisternum* (see Fig. 6). Characters of the median lobe and the parameres are diagnostic at the species level.

#### Key to the species of *Crucisternum*

**Table:** 

1	Elytra with ground punctures rather sharply marked	**2**
–	Elytra with ground punctures shallowly marked	**5**
2	Aedeagus fusiform; gonopore on apical region of median lobe; aedeagus widest near midlength (Fig. 6A–C)	**3**
–	Aedeagus pear-shaped; gonopore located near midlength of median lobe; aedeagus widest near basal third (e.g., Fig. 6D–J)	**4**
3	Apical third of parameres nearly parallel-sided; outer margins of parameres slightly sinuate along apical half	***C. ouboteri***
–	Apical third of parameres gradually narrowing towards apex; outer margins of parameres nearly straight along apical half	***C. toboganensis***
4	Apical third of median lobe parallel-sided, nearly as wide as a paramere at the base of its apical third; apex of parameres pointing inwards (Fig. 6D)	***C. vanessae***
–	Apical third of median lobe with converging margins, narrower than a paramere at the base of its apical third; apex of parameres pointing outwards (Fig. 6J)	***C. queneyi***
5	Outer margins of parameres widely curved, at most slightly sinuated; median lobe constricted at midlength, continuing as a narrow and roundly pointed bar (Fig. 6I)	***C. escalera***
–	Outer margins of parameres abruptly constricted at apical third, or nearly straight to sinuated only if median lobe gradually narrowing towards apex (Fig. 6E–H)	**6**
6	Median lobe constricted at apical third, continuing as a narrow and roundly pointed bar; outer margins of parameres abruptly constricted at apical third, continuing nearly parallel towards apex; concavity of inner margins of parameres extending only along apical sixth (Fig. 6G, H)	***C. sinuatus***
–	Median lobe gradually narrowing towards apex; apex of median lobe rounded; outer margins of parameres sinuate to nearly straight; concavity of inner margins of parameres extending along apical third (Fig. 6E, F)	***C. xingu***

### Crucisternum escalera sp. n.

Taxon classificationAnimaliaColeopteraHydrophilidae

http://zoobank.org/8C7098E9-13C8-4EEE-88FA-C548CEB1BA23

**Figure 8.**
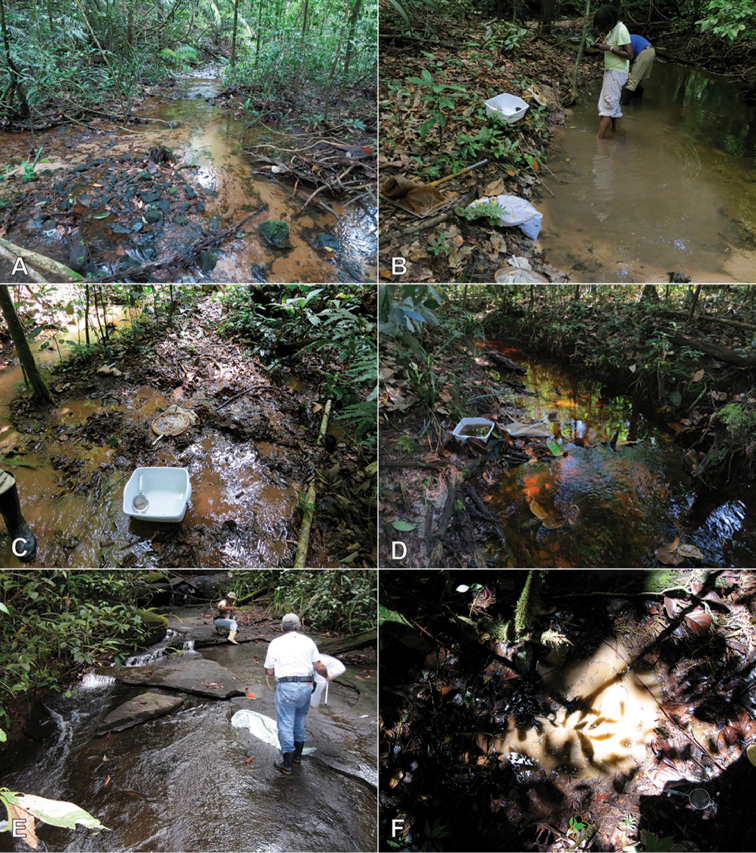
Habitat of *Crucisternum* spp. **A–B** Habitat for *C. ouboteri* Suriname, Raleighvallen, collecting event SR12-0727-03A **C** Type locality and habitat for *C. ouboteri*, Suriname, Brownsberg Nature Park, collecting event SR12-0804-01A **D** Type locality and habitat for *C. vanessae*, Suriname, Tafelberg Summit, Caiman Creek, collecting event SR13-0819-05D **E–F** Type locality and habitat for *C. escalera*, Venezuela, Bolivar State, along La Escalera, collecting event AS-08-058.

Figs 1A–D ; 6I ; 7 ; 8E, F

#### Type material examined.

**Holotype (male)**: “**VENEZUELA**: Bolívar State/ 6°2'10.5"N, 61°23'57.8"W, 630 m/ Along La Escalera; 31.vii.2008/ leg. A. Short, M. García, L. Joly/ AS-08-058; rocky stream” // “Barcode/ SM00828756/ KUNHM-ENT” (MIZA). **Paratypes (2)**: Same data as holotype (SEMC, 2).

#### Differential diagnosis.

*Crucisternum escalera* is very similar to *C. sinuatus* and *C. xingu* in the shallowness of the punctation and the uniform coloration along the body. It shares with *C. sinuatus* the shape of the median lobe (constricted at midlength, continuing as a narrow and roundly pointed bar, see Fig. 6 G–I), but can be distinguished from it by the outer margins of the parameres, which are widely curved, to at most slightly sinuate (see Fig. 6I; as opposed to strongly constricted near midlength, Fig. 6G, H).

#### Description.

Body length 2.1–2.2 mm, width 1.1–1.2 mm. General coloration uniformly orange brown (Fig. 1 A–D). Elytra with punctures shallowly marked. Aedeagus (Fig. 6I) with outer margins of parameres widely curved, at most slightly sinuated; median lobe strongly constricted near midlength, continuing as a narrow and roundly pointed bar; gonopore located at midlength of narrow apical half of median lobe.

#### Etymology.

Noun in apposition. Named after the type locality of “La Escalera”, which is the road that ascends from lowlands to the high-elevation plateau known as the “Gran Sabana”.

#### Distribution.

Venezuela (Bolívar). Collected at 630 m in elevation. See Fig. 7.

#### Biology.

The only known series of this species was collected along the margin of a densely forested rocky stream. See Fig. 8E, F.

### Crucisternum ouboteri sp. n.

Taxon classificationAnimaliaColeopteraHydrophilidae

http://zoobank.org/AD02C73E-2E54-4352-96C4-584A5D6B6982

Figs 2A–D ; 4 ; 5A, B ; 6A, B ; 7 ; 8A–C

#### Type material examined.

**Holotype (male)**: “**SURINAME**: Sipaliwini district/ 04°56.871'N, 55°10.911'W, 462 m/ Brownsberg Nature Park, forested/ stream with lots of detritus; leg./ Short, Maier, McIntosh; 4.viii.2012/ SR12-0804-01A” // “Barcode/ SEMC1113824/ KUNHM-ENT” (NZCS). **Paratypes (155): GUYANA: Region IX**: “2°06.492'N, 59°13.653'W, 274 m/ Parabara, N. side of river/ small flowing forested creek/ detritus margins & leaf packs/ leg. Short, Isaacs, Salisbury/ 3.xi.2013; GY13-1103-02A” (5, SEMC). **FRENCH GUIANA: Roura**: “[Patawa: crique/ Diamant]/ 16.xi.2007/ P. Queney réc.” (1, PQPC). **SURINAME: Sipaliwini district**: “2°21.776'N, 56°41.861'W, 237 m/ Camp 3, Wehepai; leg. Short &/ Kadosoe; sandy forest creek/ 4–6.ix.2010; SR10-0904-01A/ 2010 CI-RAP Survey” (2, SEMC); “2.97731°N, 55.38500°W, 200 m/ Camp 4 (low), Kasikasima; sandy stream on trail to METS camp/ 20.iii.2012; SR12-0320-02A/ leg. A. Short; 2012 CI-RAP Survey” (27, SEMC, including DNA voucher SLE 472); same, except “sandy creek, trail to Kasikasima/ 22.iii.2012; SR12-0322-02A” (12, SEMC); “04°42.480'N, 56°13.159'W, 24 m/ Raleighfallen Nature Reserve, trail/ to Raleighfallen; creek margins/ leg. Short, McIntosh, & Kadosoe/ 27.vii.2012; SR012-0727-03A” (1, SEMC); same data as holotype (89, NZCS, SEMC, MALUZ). **VENEZUELA: Amazonas**: “Cerro de la Neblina, basecamp 140m./ 0°50'N, 66°10'W/ 18 February 1985// Berlese of leaf packs/ among rocks in small/ stream in rainforest/ P.J. & P.M. Spangler, R. Faitoute, W. Steiner” (16, USNM, MIZA); same, except “rainforest at black light/ 21.ii.1985; leg. Spangler et al.” (1, USNM).

#### Differential diagnosis.

*Crucisternum ouboteri* is very similar to *C. vanessae* and *C. queneyi* in the sharpness of the punctation (compare Fig. 2A–D to 2E–H and Fig. 1E–H). It is nearly indistinguishable from *C. toboganensis* based on external characters beyond coloration (uniform along the body in *C. toboganensis*, paler pronotum in *C. ouboteri*). *Crucisternum ouboteri* can be recognized by the fusiform aedeagus, with the apical third of the parameres parallel-sided with sinuate outer margins (Fig. 6A, B).

#### Description.

Body length 2.0–2.4 mm, width 1.2–1.4 mm. General coloration dark brown on elytra, slightly paler on pronotum and head (Fig. 2A). Elytra with punctures rather sharply marked. Aedeagus fusiform, widest near mid length; median lobe gradually narrowing from basal fifth towards apical region; gonopore usually presented longitudinally on apical region of median lobe; apical third of parameres parallel-sided, with sinuate outer margins (Fig. 6A, B).

#### Etymology.

Named in honor of Dr. Paul Ouboter, director of the National Zoological Collection of Suriname and leading authority on the biodiversity of Suriname. Dr. Ouboter’s assistance has been invaluable in our efforts to document the water beetle fauna of the country.

#### Distribution.

French Guiana, Guyana, Suriname, and Venezuela (Amazonas). See Fig. 7. Collected at 24 – 462 m elevation.

#### Biology.

This species is known from densely forested streams throughout much of the Guiana Shield region of South America (e.g., Fig. 8A–C). One specimen was collected at a UV light in southern Venezuela.

### Crucisternum queneyi sp. n.

Taxon classificationAnimaliaColeopteraHydrophilidae

http://zoobank.org/00079818-D26E-4DBE-862B-C78C7D6F6D99

Figs 1E–H ; 6J ; 7

#### Type material examined.

**Holotype (male): FRENCH GUIANA: Sinnamary**: “[Pripris de Yiyi]/ 25.xi.2007/ P. Queney réc.” (MNHN). **Paratypes (2): FRENCH GUIANA: Roura**: “[Patawa: ru]/ 19.xi.2007/ P. Queney réc.” (1, SEMC); “[N2, PK 73,5: petite crique accidentée, 250m]/ 23.xi.2007/ P. Queney réc.” (1, PQPC).

#### Differential diagnosis.

*Crucisternum queneyi* is very similar to *C. ouboteri* and *C. vanessae* in the sharpness of the punctation and the general coloration of the body (compare Fig. 1E–H to Fig. 2). It can be readily recognized by the pear-shaped aedeagus (as opposed to fusiform), with simple (as opposed to broadened) margins of the apical region of the median lobe, as well as outwardly pointing apices of the parameres (see Fig. 6J).

#### Description.

Body length 1.9–2.3 mm, width 1.0–1.1 mm. General coloration dark brown on elytra, slightly paler on pronotum and head (Fig. 1E). Elytra with punctures rather sharply marked. Aedeagus pear-shaped, widest along basal third; apex of parameres pointing outwards; apical third of median lobe narrower than a paramere at the base of its apical third, with simple converging margins; gonopore located near midlength of median lobe (Fig. 6J).

#### Etymology.

Named after French coleopterist Pierre Queney, who collected and beautifully prepared the known specimens of this species.

#### Distribution.

French Guiana (Roura). See Fig. 7. Collected at 250 m elevation.

#### Biology.

This species is known from streams.

### Crucisternum sinuatus sp. n.

Taxon classificationAnimaliaColeopteraHydrophilidae

http://zoobank.org/BD20616D-2360-4509-84FE-210B65E9D54E

Figs 3A–D ; 6G, H ; 7

#### Type material examined.

**Holotype (male): BRAZIL: Minas Gerais**: Lassance, -17.83384, -44.50515, 568 m, Cachoeira da Palmeira, flotation of root mats and moss from side of waterfall and seepage, 2.iii.2018, leg. Benetti & team; BR18-0302-04A (INPA). **Paratypes (10): BRAZIL: Pará**: Rio Xingu Camp 52°22'W, 3°39'S, Altamira, ca 60km S., 3.x.1986, leg. P. Spangler & O. Flint, Colln. #6, 1^st^ jungle stream on trail 1 (1, USNM); same, except 14.x.1986, Colln. #23, stream on left branch of trail 1 (3; SEMC, USNM); **Minas Gerais**: same data as holotype (2 INPA, 4 SEMC).

#### Differential diagnosis.

*Crucisternum sinuatus* is very similar to *C. escalera* and *C. xingu* in the shallowness of the punctation. In addition, it can be distinguished from *C. xingu* by the aedeagus with median lobe constricted at midlength, continuing as a narrow and roundly pointed bar (see Fig. 6G, H; as opposed to gradually narrowing towards the apex, see Fig. 6E, F), and the outer margins of the parameres, which are strongly constricted at apical third, continuing nearly parallel (as opposed to nearly straight to sinuate).

#### Description.

Body length 2.0–2.4 mm, width 1.1–1.4 mm. General coloration variable, either orange brown or uniformly dark brown (Fig. 3A–D). Elytra with punctures shallowly marked. Aedeagus with outer margins of parameres strongly constricted at apical third, continuing nearly parallel towards apex; inner margins of parameres slightly concave only along apical sixth; median lobe constricted at midlength, continuing as a narrow and roundly pointed bar (Fig. 6G, H).

#### Etymology.

Named in reference to the abrupt constriction of the outer margins of the parameres at their apical third, with the Latin word *sinuatus* meaning bent, curved.

#### Distribution.

Brazil (Minas Gerais, Pará). See Fig. 7.

#### Biology.

The series from Lassance was collected by floating root mats and detritus that were on rock at the margin of a river and waterfall. The specimens from Altamira were collected in streams.

### Crucisternum toboganensis sp. n.

Taxon classificationAnimaliaColeopteraHydrophilidae

http://zoobank.org/D6CCF6F9-9452-4074-A71C-B8891F894008

Figs 6C ; 7

#### Type material examined.

**Holotype (male): VENEZUELA: Amazonas**: “Puerto Ayacucho (40 km S)/ El Tobogán, Caño Coromoto/ 26 January 1989/ CL2388, John T. Polhemus/ side stream/ head of lower falls” (USNM) **Paratypes (2)**: Same data as holotype (1, USNM); same locality but leg. M. Balke (1, SEMC; DNA voucher SLE 734).

#### Differential diagnosis.

*Crucisternum toboganensis* is nearly indistinguishable from *C. ouboteri* based on external characters beyond coloration (uniform along the body in *C. toboganensis*, paler pronotum in *C. ouboteri*). They can be recognized by characters of the aedeagus: the apical third of the parameres gradually narrow towards the apex, with straight outer margins in *C. toboganensis* (Fig. 6C), whereas in *C. ouboteri* the apical third of the parameres is parallel-sided and has sinuate outer margins (Fig. 6A, B).

#### Description.

Body length 2.1–2.4 mm, width 1.2–1.3 mm. General coloration uniformly brown along body regions. Elytra with punctures rather sharply marked. Aedeagus (Fig. 6C) fusiform, widest slightly beyond mid length; median lobe gradually narrowing from basal fifth towards apical region; gonopore on apical region of median lobe; apical third of parameres gradually narrowing towards apex, with straight outer margins.

#### Etymology.

Named after the type locality: El Tobogán de la Selva in Venezuela.

#### Distribution.

Venezuela (Amazonas). See Fig. 7.

#### Biology.

Nothing is known about the biology of this species except that it was collected from the margin of the Rio Coromoto, the infamous locality for many water beetle species including the family Meruidae (see Spangler and Steiner 2005).

### Crucisternum vanessae sp. n.

Taxon classificationAnimaliaColeopteraHydrophilidae

http://zoobank.org/1AF57F85-A0E3-4F1E-B524-089391139907

Figs 2E–H ; 6D ; 7 ; 8D

#### Type material examined.

**Holotype (male)**: “**SURINAME**: Sipaliwini district/ 3°53.942’N, 56°10.849’W, 733 m/ CSNR: Tafelberg Summit, near/ Caiman Creek Camp, margins & leaf packs in Caiman Creek/ leg. Short & Bloom; 19.viii.2013/ SR13-0819-05D” (NZCS). **Paratypes (188): SURINAME: Sipaliwini district**: “3°55.600’N, 56°11.300’W, 600 m/ CSNR: Tafelberg Summit/ Augustus Creek, seepage on/ wall; leg. Short & Bloom/ 14.viii.2013; SR13-0814-01B” (3, SEMC); “3°56.351’N, 56°10.954’W, 614 m/ CSNR: Tafelberg Summit/ Geijskes Creek, margin of creek/ w/ detritus; leg. Short & Bloom/ 15.viii.2013; SR13-0815-02A” (24, NZCS, SEMC); same, except “seep on wall/ 16.viii.2013; SR13-0816-01A” (1, SEMC); same, except “leaf packs & wood jams/ SR13-0816-01B” (4, SEMC); same, except “leaf packs & rock scrubbing; leg. D. Bloom/ SR13-0816-01D” (13, SEMC); “3°55.600’N, 56°11.300’W, 600 m/ CSNR: Tafelberg Summit, nr/ Augustus Creek Camp pools &/ creeks on trail into Arrowhead/ basin; leg. Short & Bloom/ 17.viii.2013; SR13-0817-01A” (3, SEMC); “3°55.600’N, 56°11.300’W, 600 m/ CSNR: Tafelberg Summit/ nr Augustus Creek Camp/ 17.viii.2013; SR13-0817-03A” (26, NZCS, SEMC); “3°53.942’N, 56°10.849’W, 733 m/ CSNR: Tafelberg Summit, near/ Caiman Creek Camp, stream/ margins; leg. Short & Bloom/ 18.viii.2013; SR13-0818-01A” (11, SEMC); same, except “forest detrital pools; SR13-0818-02A” (1, SEMC); same, except “pools in forest/ 19.viii.2013; SR13-0819-05B” (8, SEMC); same, except “margins & leaf packs in Caiman Creek/ SR13-0819-05D” (79, MIZA, MALUZ, SEMC); “3°55.600’N, 56°11.300’W, 600 m/ CSNR: Tafelberg Summit, near/ Augustus Creek Camp, detrital/ creek; leg. Short & Bloom/ 22.viii.2013; SR13-0822-01A” (14, SEMC); same, except “leaf packs/ SR13-0822-03A” (1, SEMC).

#### Differential diagnosis.

*Crucisternum vanessae* is very similar to *C. ouboteri* and *C. queneyi* in the sharpness of the punctation and the general coloration of the body (compare Fig. 2E–H to 2A–D and Fig. 1E–H, respectively). It can be readily recognized by the pear-shaped aedeagus (as opposed to fusiform), with the apical third of the median lobe parallel-sided, as well as inwardly pointing apices of the parameres (Fig. 6D).

#### Description.

Body length 2.0–2.5 mm, width 1.2–1.4 mm. General coloration dark brown on elytra, slightly paler on pronotum and head (Fig. 2E). Elytra with punctures rather sharply marked. Aedeagus (Fig. 6D) pear-shaped, widest at basal third; apex of parameres slightly pointing inwards; apical third of median lobe parallel-sided, nearly as wide as a paramere at the base of its apical third; gonopore located at apical third of median lobe.

#### Etymology.

Named after Surinamese entomologist Vanessa Kadosoe who has assisted us on numerous expeditions in Suriname, including the expedition to Tafelberg on which this species was discovered.

#### Distribution.

Currently known only from several streams on the summit of Tafelberg Tepui in central Suriname. Collected at elevations from 600 to 733 m. See Fig. 7.

#### Biology.

This species was collected in several forested streams that drain the summit of a low-elevation tepui. Specimens were abundant in several spots, usually where lots of fallen leaves and detritus had accumulated along the stream margins. Specimens were collected by submerging the leaf packs and catching the specimens that floated to the water surface. See Fig. 8D.

### Crucisternum xingu sp. n.

Taxon classificationAnimaliaColeopteraHydrophilidae

http://zoobank.org/7EF9B5FF-DD04-423E-80F8-CDF21AE0DD1A

Figs 3E–H ; 6E, F ; 7

#### Type material examined.

**Holotype (male)**: “**BRAZIL**: Pará/ Rio Xingu Camp 52°22'W, 3°39'S/ Altamira, ca 60km S.; 14.x.1986/ leg. P. Spangler & O. Flint// Colln. #23, stream on/ left branch of trail 1” (USNM). **Paratype (1): BRAZIL: Pará**: same, except “3.x.1986; Colln. #6;/ 1^st^ jungle stream on trail 1” (1, USNM).

#### Differential diagnosis.

*Crucisternum xingu* is very similar to *C. sinuatus* and *C. escalera* in the shallowness of the punctation and the uniform coloration along the body. It can be readily recognized by its dark brown coloration (as opposed to orange brown; compare Fig. 3E–H to Fig. 1), which is shared with *C. sinuatus*, from which it can be distinguished by the median lobe gradually narrowing towards the apex (see Fig. 6E, F; as opposed to constricted at midlength, continuing as a narrow and roundly pointed bar, see Fig. 6G, H), and the nearly straight to sinuate outer margins of the parameres (as opposed to strongly constricted at apical third, continuing nearly parallel).

#### Description.

Body length 2.2–2.4 mm, width 1.3–1.4 mm. General coloration uniformly dark brown (Fig. 3E). Elytra with punctures shallowly marked. Aedeagus with outer margins of parameres sinuate to nearly straight; inner margins of parameres concave along apical third; median lobe gradually narrowing towards apex; apex of median lobe rounded (Fig. 6E–F).

#### Etymology.

Noun in apposition. Named after the Xingu River where the known specimens were collected.

#### Distribution.

Brazil (Pará). See Fig. 7.

#### Biology.

This species is known from forest streams.

### Crucisternum

Taxon classificationAnimaliaColeopteraHydrophilidae

sp.

#### Material examined

**(8). BRAZIL: Pará**: “Rio Xingu Camp 52°22'W, 3°39'S/ ca 60km S. of Altamira; 3.x.1986/ leg. P. Spangler & O. Flint; Colln. #6;/ 1^st^ jungle stream on trail 1” (6; USNM; unassociated females; likely either *C. sinuatus* or *C. xingu*); **Roraima**: Amajari: Serra do Tepequém, Igarape Preto Negro, Quebrada do Canara, 3°46.715'N, 61°45.405'W, 637 m, 14.i.2018, forested stream, Short, Benetti & Santana, BR18-0114-05A (1, SEMC). **FRENCH GUIANA: Roura**: “[Patawa: crique/ Diamant]/ 16.xi.2007/ P. Queney réc.” (1, SEMC; unassociated female; likely either *C. ouboteri* or *C. queneyi*); “[Patawa: ru]/ 19.xi.2007/ P. Queney réc.”; “[N2, PK 73,5: petite crique accidentée, 250m]/ 23.xi.2007/ P. Queney réc.” (1, SEMC; unassociated female; likely either *C. ouboteri* or *C. queneyi*). **SURINAME: Sipaliwini district**: “3°47.479’N, 56°08.968’W, 320 m/ CSNR: near Kappel airstrip/ at Tafelberg trail, in creek/ leg. Short & Bloom; 13.viii.2013; SR13-0813-06B” (1, SEMC; unassociated female; likely either *C. ouboteri* or *C. vanessae*).

#### Remarks.

Given our current inability to identify some species based only on external characters, these unassociated females could not be identified with confidence, especially in the localities where more than one species are known to occur.

### Katasophistes gen. n.

Taxon classificationAnimaliaColeopteraHydrophilidae

http://zoobank.org/3EB28C66-5B17-4540-8E86-DB99504C7227

**Figure 9.**
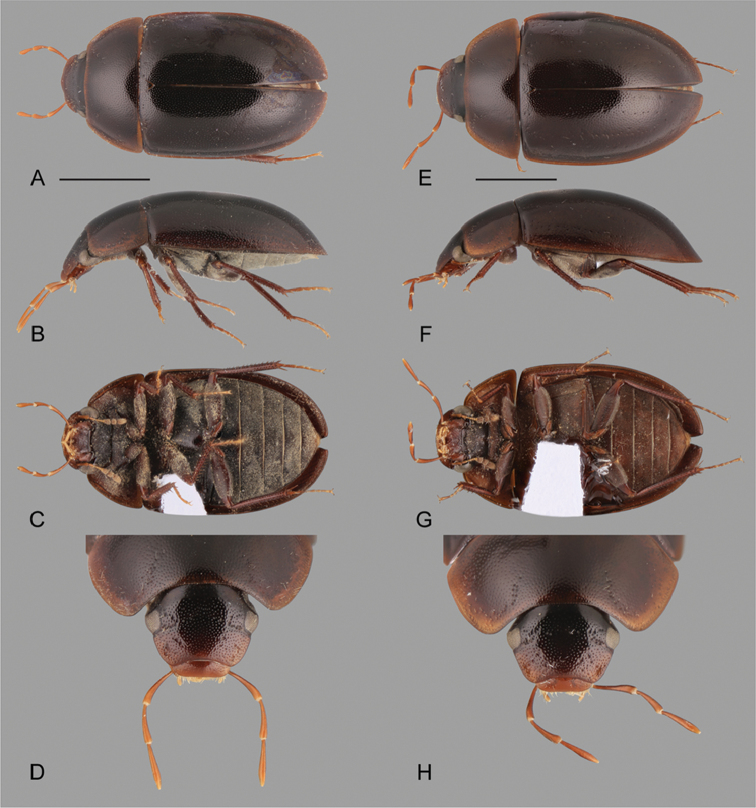
Habitus of *Katasophistes* spp.: **A–E** *Katasophistes charynae*: **A** dorsal view **B** lateral view **C** ventral view **D** head **E–H** *Katasophistes cuzco*: **E** dorsal view **F** lateral view **G** ventral view **H** head. Scale bars: 1 mm.

**Figure 10.**
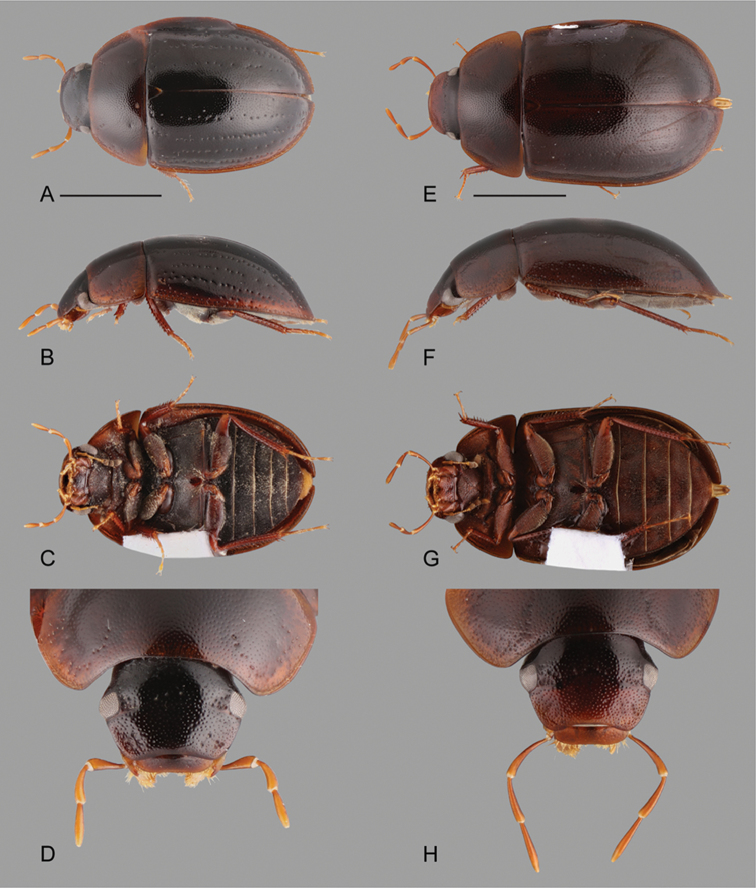
Habitus of *Katasophistes* spp.: **A–E** *Katasophistes merida*: **A** dorsal view **B** lateral view **C** ventral view **D** head **E–H** *Katasophistes superficialis*: **E** dorsal view **F** lateral view **G** ventral view **H** head. Scale bar: 1 mm.

**Figure 11.**
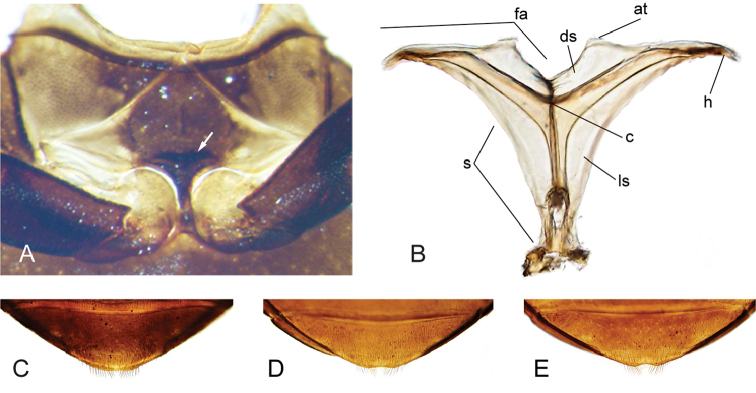
Thorax and abdomen of *Katasophistes* spp.: **A–C** *Katasophistes merida*: **A** ventral view of mesoventrite (white arrow pointing transverse ridge) **B** posterior view of metafurca: (at) anterior tendon, (c) crux, (ds) dorsal sheath, (fa) furcal arm, (h) hemiductus, (ls) lateral sheath, (s) stalk **C** fifth abdominal ventrite **D** *Katasophistes charynae*, fifth abdominal ventrite; **E** *Katasophistes cuzco*, fifth abdominal ventrite.

**Figure 12.**
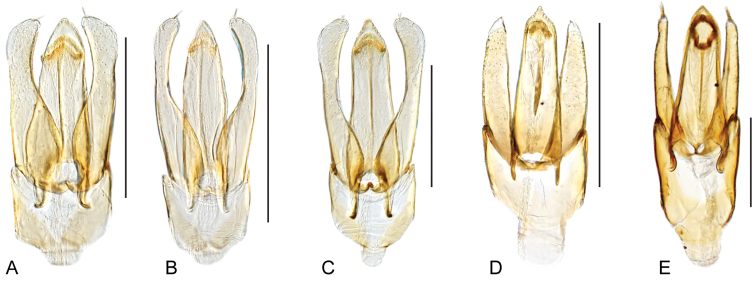
Aedeagus of *Katasophistes* spp.: **A–B** *Katasophistes charynae*: **A** “colln 32” **B** “colln 42a” **C** *Katasophistes cuzco*; **D** *Katasophistes merida* **E** *Katasophistes superficialis*. Scale bars: 0.3 mm.

**Figure 13.**
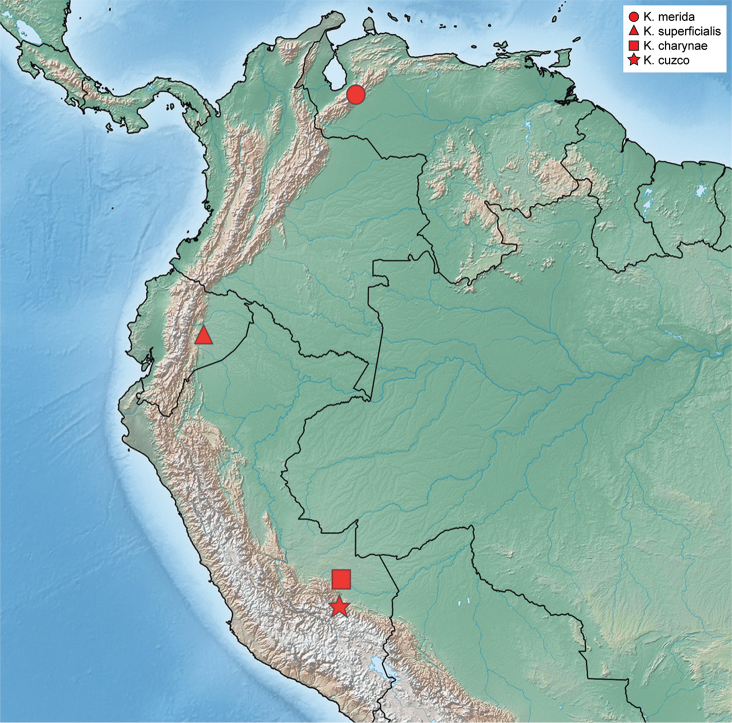
Distribution of *Katasophistes* species.

**Figure 14.**
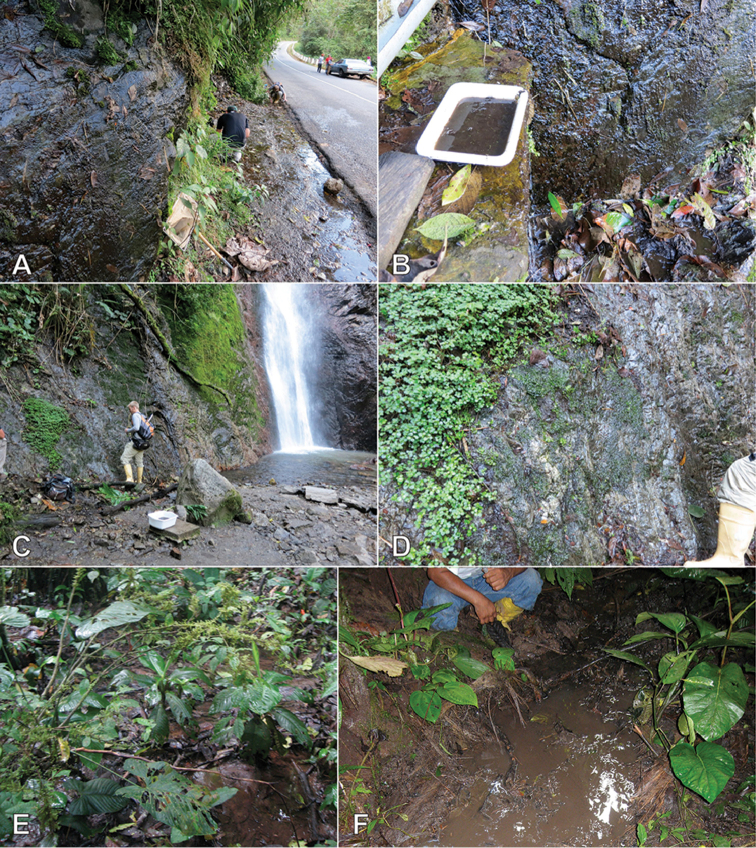
Habitat of *Katasophistes* spp. **A–B** Type locality and habitat of *K. merida*: Venezuela: Merida State, Collecting Event VZ12-0122-03A **C–D** Habitat of *K. merida*: Venezuela: Merida State, Collecting Event VZ12-0124-01A **E–F** Type locality and habitat of *K. superficialis*: Ecuador: Pastaza Province, Collecting Event AS-08-08b.

Figs 9 , 10 , 11 , 12 , 13 , 14

#### Type species.

*Katasophistes merida*

#### Differential diagnosis.

Medium to small beetles, total body length 2.7–4.5 mm, width 1.7–2.4 mm. Color orange brown to dark brown, rather uniform along body regions. Body shape oval to elongated in dorsal view; moderately convex in lateral view (see Figs 9–10). Antennae with nine antennomeres (e.g., Fig. 10C). Maxillary palps curved inward, moderately long to long (e.g., Fig. 10D, H). Each elytron with five rows of deep/large systematic punctures (Fig. 10A; not as evident in *K. charynae*, *K. cuzco* and *K. superficialis*); elytra without sutural striae, with outer margins slightly flared; serial punctures not evident. Posterior elevation of mesoventrite, with a well-defined, curved transverse ridge (Fig. 11A). Posterior femora glabrous at most along apical third. Fifth abdominal ventrite apically truncate to slightly emarginate, with stout setae (see Fig. 11C–E).

At first glance the genus may appear similar to some species of *Chasmogenus*, however the lack of sutural striae easily separates the two. The enlargement of the rows of elytral systematic punctures is also rare within the Acidocerinae (found in some *Chasmogenus* and *Agraphydrus*) and will separate it from other New World *Helochares*, with which it may also be confused.

#### Description.

Body oval to elongate, weakly to moderately convex (e.g., Figs 9F, 10F), coloration rather uniform along body regions, orange brown to dark brown, often with maxillary palps slightly paler. **Head.** Frons and clypeus (e.g., Fig. 9D) with moderately marked ground punctures, irregularly and rather densely distributed over the surface, accompanied by seta-bearing systematic punctures, particularly noticeable on frons behind frontoclypeal suture and around inner margins of eyes, and on lateral areas of clypeus; surface between punctures smooth and shiny; anterior corners of clypeus roundly angulated; anterior margin of clypeus widely roundly emarginate. Eyes subquadrate in dorsal view (e.g., Fig. 10H). Labrum wide, fully exposed, nearly half as long, and collinear to clypeus (e.g., Fig. 9D); dorsal surface slightly convex, with scattered fine punctures; anterior margin mesally slightly emarginate and barely bent inwards; lateral and anterior margins fringed by rather long setae. Temporae densely covered by very short and fine setae (hydrofuge pubescence). Mentum parallel sided, with lateral oblique longitudinal ridges; anterior margin with wide, deep, concave median impression, not demarcated by a transverse carina. Submentum rather flat and pubescent at base, glabrous, shiny and ascending at apex, with sparse rather long setae near limit of pubescent/glabrous line; ocular ridge well-developed (e.g., Fig. 9C). Maxilla (e.g., Fig. 9C) with ventral surface of cardo and stipes smooth and shiny, with scattered and shallow punctures (basal outer margin of cardo with a fringe of fine and rather long setae in *K. merida*); outer dorsal margin of palpifer with a row of stiff decumbent spiniform setae; limit between cardo and stipes oblique; maxillary palps brown to orange, slender, longer than antennae (slightly so and stout in *K. merida*); apex of palpomere 3 bearing sensilla. Mandibles with apex bifid (observed in *K. merida* and *K. charynae*). Labial palps yellowish, nearly as long as mentum, dorsoventrally flattened; palpomere 2 with inner and outer margin convex apicad of midpoint; palpomere 3 digitiform, with a long subapical seta on outer corner. Antennae (e.g., Fig. 10C) with nine antennomeres, either similar or paler than general coloration of head; antennomere 1 anteriorly projected near base, almost reaching midpoint of ventral surface of eye (not reaching cardo-stipes joint), nearly 1.4–1.8-times longer than antennomere 2; antennomere 2 nearly as long as antennomeres 3–4 combined; antennomere 6 forming a well differentiated, only slightly asymmetric cupule; antennomeres 7–9 similar in size (8 shortest, 9 longest) and shape, slightly flattened (less so in *K. merida*), forming a loosely articulated, pubescent club; apex of antennomeres 7–9 with few scattered setae longer than general pubescence of club. **Thorax.** Pronotum widest at base, narrowed anteriorly, with anterior and posterior margins only slightly sinuate; surface evenly convex, ground punctation moderate, uniformly dense, with surface between punctures smooth and shiny; seta bearing systematic punctures forming paired anterolateral semicircles. Scutellar shield of moderate size, triangular, nearly as long as wide, with punctation as in pronotum. Prosternum (e.g., Fig. 9C) nearly as long as 2/3 of length of a coxa, weakly convex to only slightly ascending longitudinally on central area; anterior margin of prosternum mesally projected as a wide, roundly pointed triangle, usually with a low carina along midline of projection; surface of prosternum covered by rather long, fine, scattered setae; intercoxal process projected from posterior margin of procoxal cavities, rectangular shaped in outline, with pyramidal surface. Mesoventrite not fused to mesepisterna, with anterior margin 0.2-times as wide as anterior margin of mesepisternum; anterior rib of mesoventrite bearing a medial semitriangular, pearlescent macula; posterior elevation of mesoventrite with a well-defined, curved transverse ridge, sometimes swollen at midpoint (as in *K. cuzco*); surface of mesoventrite reticulated for the most part; mesepisternum obliquely widely concave, with reticulated surface; mesepimeron trapezoid, with reticulate and pubescent surface. Mesofurca (examined in *K. merida*) with short arms, 0.6-times length of mesocoxae; apical half of arms free irregularly explanate, with outer corners sharply pointed. Metaventrite posteromesally elevated, with elevation rather flat; surface of metaventrite reticulate and pubescent, except for postero median and glabrous patch. Metepisterna 3–4-times longer than wide, only slightly narrowing posteriorly. Metepimeron triangular and acute. Metafurca (examined in *K. merida*, Fig. 11B) 1.6-times wider than long, with furcal arms (fa) slightly longer than stalk (s); stalk triangular (wider near the crux (c), gradually narrowing ventrally), with paired longitudinal keels extending along basal third of posterior face, and a well-developed median keel on anterior face extending to anterior margin of dorsal sheets (ds); outer margins of stalk diverging from base towards proximal third of furcal arms; furcal arms somewhat trapezoid, with apex (hemiductus (h)) only slightly explanate, obliquely positioned; anterior tendons (at) inserted at basal third of dorsal edge of furcal arms; dorsal sheaths well-developed, slightly wider than widest point of lateral sheaths (ls). **Elytra.** Surface even (without elevations or depressions), serial punctures not clearly defined (e.g., Fig. 9E), not impressed into striae; seta bearing systematic punctures rather enlarged and deep (less strikingly so in *K. charynae*, *K. cuzco* and *K. superficialis*), forming five longitudinal rows, fifth row very close to outer margin of elytron; elytral margins slightly flared. Epipleura well-developed, slightly oblique, with sparse setae, anteriorly wide, gradually narrowing posteriorly, extending up to line of posterior margin of first abdominal ventrite; pseudepipleura well-developed, perpendicularly positioned, nearly half as wide as anterior portion of epipleura, extending along entire outer margin of elytra. Hind wings well-developed. **Legs.** All femora with dense pubescence, at least along basal two thirds, remainder of surface glabrous, shiny and slightly reticulated; all femora antero-posteriorly flattened; metafemora with rather weak tibial grooves. Tibiae slender, weakly flattened, with well-developed spines; protibiae with a median longitudinal row of long spathulate setae along anterior surface. All tarsi with five tarsomeres, bearing long apical hair-like setae on dorsal face, and two lateral rows of spines and/or hair-like spines on ventral face of tarsomeres 2–4; protarsomeres 1–4 similar in size and shape; meso- and meta tarsomeres with tarsomere 2 nearly as long as tarsomere 5; tarsomere 5 approximately as long as tarsomeres 3–4 combined, without spines on ventral face; claws rather large, curved; empodium well-developed, bearing a pair of long, curved apical setae. **Abdomen.** Abdomen with five ventrites, medially weakly convex; all ventrites reticulated, with uniform, dense, fine pubescence; posterior margin of fifth ventrite truncate to mesally weakly emarginated, set with a row of thick, flat spine-like setae (see Fig. 11C–E). Aedeagus (Fig. 12) nearly parallel sided, with basal piece between 0.5 and 1.1-times the length of parameres; median lobe wider than each paramere, gradually narrowing apically, with a conspicuous median longitudinal sclerotization, and well-developed lateral basal apodemes; apex of median lobe acute; parameres nearly as long as median lobe, with apical setae; gonopore preapically situated.

#### Larvae.

The immature stages are unknown.

#### Etymology.

Named from the Greek word *katasophistes*, meaning trickster, in reference to the disparity of the general appearance of some of the known species. Name to be treated as masculine.

#### Distribution.

Venezuela (Mérida), Ecuador (Pastaza), Peru (Cuzco, Madre de Dios). See Fig. 13.

#### Biology.

Species in this genus exhibit a combination of ecologies: *K. merida* is restricted to seepage habitats, while the type series of *K. superficialis* was collected from forested stream pools with abundant detritus. See Fig. 14.

#### Characters of taxonomic importance for *Katasophistes*.

With the exception of the enlarged elytral systematic punctures, there is nothing particularly remarkable about the external morphology of *Katasophistes*. Careful examination of the elytral systematic punctures is needed in order to recognize *K. charynae*, *K. cuzco*, and *K. superficialis*.

***Punctation*.** The most prominent feature of *Katasophistes* is the enlargement of the elytral systematic punctures, which is evident in *K. merida* (Fig. 10A, B), but much less so in *K. charynae*, *K. cuzco* and *K. superficialis*. One way to recognize the enlarged elytral systematic punctures would be by spotting the long setae that systematic punctures bear.

***Posterior elevation of mesoventrite*.** It is usually well-defined and shaped as a curved transverse ridge. Only in *K. cuzco* is this transverse ridge additionally medially swollen, as to form a low bump.

***Aedeagus*.** In *Katasophistes* the aedeagus exhibits the same general shape of the median lobe, combined with a wide variety of shapes of the parameres (see Fig. 12).

#### Key to the species of *Katasophistes*

**Table:** 

1	Elytra with strikingly large and deeply impressed systematic punctures (Fig. 10A, B); maxillary palps relatively short and stout (Fig. 10D)	***K. merida***
–	Elytra with slightly enlarged systematic punctures which are shallowly impressed; maxillary palps relatively long and slender (e.g., Fig. 10H)	**2**
2	Body length around 4.5 mm; basal piece of aedeagus nearly as long as parameres (Fig. 12E)	***K. superficialis***
–	Body length less than 4 mm; basal piece of aedeagus nearly half as long as parameres (Fig. 12A–C)	**3**
3	Transverse ridge on posterior elevation of mesoventrite uniformly wide; parameres with widest basal point clearly wider than pre-apical width (Fig. 12A, B)	***K. charynae***
–	Transverse ridge on posterior elevation of mesoventrite swollen at midpoint; parameres with widest basal point nearly as wide as pre-apical width (Fig. 12C)	***K. cuzco***

### Katasophistes charynae sp. n.

Taxon classificationAnimaliaColeopteraHydrophilidae

http://zoobank.org/CF86E4B5-FA2D-4CA8-B17B-AB5F0114F7DF

Figs 9A–D ; 11D ; 12A, B ; 13

#### Type material examined.

**Holotype (male)**: “**PERU: Madre de Dios**: Parque Manu, Pakitza/ 12°07'S 70°58'W/ R.A. Faitoute, colln 32/ 250 m, stream/ Trocha Dos, c72/ 10 Sept 1989” (USNM). **Paratypes (39): PERU: Madre de Dios**: Same data as holotype (14, SEMC, USNM); same, except “colln 32a, berlesed leaf litter” (12, SEMC, USNM); same, except “11–15 Sept 1989, colln 35a, Trocha uno, c 11, flight intercept trap in dry narrow stream bed” (2, USNM); same, except “16 Sept 1989, colln 42a, Trocha Dos, c.53, berlesed leaf litter” (2, USNM), same, except “18 Sept 1989, colln 46, Trocha Dos, c 14, spring seepage” (4, USNM, SEMC); same, except “19 Sept 1989, colln 47, Trocha Uno, c 14, stream” (2, USNM); same, except “colln 47a, berlesed leaf litter” (2, USNM); same, except “22 Sept 1989, coll 55a, TC 22, berlesed leaf litter & root mats” (1, USNM).

#### Differential diagnosis.

*Katasophistes charynae* is very similar to *K. cuzco*, from which it can be distinguished by its smaller size, generally darker coloration, simple curved transverse ridge on the posterior elevation of mesoventrite and characters of the aedeagal parameres.

#### Description.

Body length 2.6 to 3.5 mm, width 1.5 to 1.9 mm. Body elongate oval, weakly convex (Fig. 9B). General coloration uniformly dark brown, with slightly paler margins of pronotum. Each elytron with five rows of shallow enlarged systematic punctures. Posterior elevation of mesoventrite with well-defined, curved, uniformly wide transverse ridge. Apex of fifth abdominal ventrite weakly emarginate (Fig. 11D). Aedeagus (Fig. 12A, B) with basal piece 0.4 to 0.5-times the length of parameres; greatest width of parameres near base, clearly wider than pre-apical width, with inner margin sinuate, at widest point, parameres nearly 0.7-times greatest width of median lobe; apex of parameres rounded, slightly widened at apex; apex of median lobe forming an acute angle.

#### Etymology.

Named after Charyn Micheli (USNM), Coleopterist and museum specialist in charge of the aquatic beetle collection (among others) at the Smithsonian Institution who has assisted the authors on numerous occasions and diligently oversees the largest water beetle collection in the world.

#### Distribution.

Peru (Madre de Dios). See Fig. 13.

#### Biology.

Most specimens were collected in streams.

#### Remarks.

There is variation in the relative proportions of the aedeagus and the shape of the parameres, with some specimens having a wider and shorter aedeagus (Fig. 12A), and some with a more slender aedeagus bearing slender parameres (Fig. 12B). As there are no external features to distinguish them, and both forms of aedeagi were found at the same localities, we refrain from calling them different species until more material and/or possibly molecular data are available.

### Katasophistes cuzco sp. n.

Taxon classificationAnimaliaColeopteraHydrophilidae

http://zoobank.org/3CF1C324-BCAB-4CB2-9475-53865C2039B3

Figs 9E–H ; 11E ; 12C ; 13

#### Type material examined.

**Holotype (male)**: “**PERU: Cuzco**: Quita Calzón, at/ km 164, 1030 m/ 13°09'S 71°22'W// 2 Sept 1989/ colln 17/ R.A. Faitoute” (USNM). **Paratypes (3): PERU: Cuzco**: Same data as holotype (2, SEMC, USNM); at Km 152 & 1 Km E. of San Pedro, 1430 m/ 13°09'S 7126'W/31 Aug 1989/ colln 10/ R.A. Faitoute” (1 male, USNM).

#### Differential diagnosis.

*Katasophistes cuzco* is very similar to *K. charynae*, from which it can be distinguished by its larger size, generally paler coloration, the medially swollen curved transverse ridge on the posterior elevation of mesoventrite, as well as characters of the aedeagal parameres.

#### Description.

Body length 3.6 to 3.7 mm, width 2.0 to 2.2 mm. Body elongate oval, moderately convex (Fig. 9F). General coloration uniformly brown, with slightly paler margins of pronotum. Each elytron with five rows of shallow enlarged systematic punctures. Posterior elevation of mesoventrite with a well-defined, curved transverse ridge, swollen at midpoint. Apex of fifth abdominal ventrite moderately emarginate (Fig. 11E). Aedeagus (Fig. 12C) with basal piece nearly 0.6-times the length of parameres; greatest width of parameres near base, nearly as wide as pre-apical width, with inner margin sinuate; at widest point, parameres nearly 0.6-times greatest width of median lobe; apex of parameres rounded, slightly widened at apex; apex of median lobe forming an acute angle.

#### Etymology.

Noun in apposition. Named after Cuzco, the Peruvian province where specimens were collected.

#### Distribution.

Peru (Cuzco). See Fig. 13.

#### Biology.

Although no biological information is listed in the label, we were able to cross reference the original field notes for these collecting events and confirm that the holotype event (“colln 17”) was made in a stream, probably from leaf packs. The paratype from “colln 10” has a combination notation of “roadside ditch” and “seep”.

### Katasophistes merida sp. n.

Taxon classificationAnimaliaColeopteraHydrophilidae

http://zoobank.org/56F9F802-091F-4404-8BAF-0B8FEB7E43F1

Figs 10A–D ; 11A–C ; 12D ; 13 ; 14A–D

#### Type material examined.

**Holotype (male)**: “**VENEZUELA**: Mérida State/ 8°51.933'N, 70°37.131'W, 1682 m/ ca. 12 km SE of Santo Domingo/ leg. Short, Arias & Gustafson, wall seeps along river; 22.i.2012; VZ12-0122-03C” (MIZA). **Paratypes: (33): VENEZUELA: Mérida State**: “12Km SE/ Sta. Domingo/II-24-1969/ P. & P. Spangler” (3, USNM); “12 Km. SE Sto. Dom./ 24 Feb. 76/ C.M. & O. S. Flint” (1, USNM); same data as holotype (14, SEMC); same data as holotype except “leg. Short, Arias, & Gustafson; log and stick jams in river; VZ12-0122-03B” (3, SEMC); same, except “Short & Arias; wall seeps; VZ12-0122-03A” (6, MIZA, MALUZ, SEMC); same, except “VZ12-0122-03C2” (1, SEMC, DNA voucher SLE 426); “8°52.423’ N, 70°37.611'W, 1616 m/ Cascada Velo de la Novia; 24.i.2012/ leg. Short, Arias, & Gustafson / seeps by waterfall/; VZ12-0124-01A” (4, SEMC); same, except “logs & Kicknetting; VZ12-0124-01B” (1, SEMC).

#### Differential diagnosis.

*Katasophistes merida* is easily recognized because of the striking rows of large and deep systematic punctures along the elytra and its relatively short and stout maxillary palps.

#### Description.

Body length 2.7–3.4 mm, width 1.7–2.4 mm. Body broadly oval, moderately convex (Fig. 10A, B). General coloration dark brown, slightly paler along margins of pronotum and elytra (Fig. 10 A, B). Each elytron with five rows of large and deep systematic punctures. Apex of fifth abdominal ventrite truncate (Fig. 11C). Aedeagus (Fig. 12D) with basal piece nearly 0.8-times the length of parameres; parameres at widest point, nearly 0.6-times greatest width of median lobe; apex of parameres narrowly round; apex of median lobe with median pointed projection.

#### Etymology.

Noun in apposition. Named after the Venezuelan State of Mérida, where the beetles have been collected.

#### Distribution.

Venezuela (Mérida). Elevation range 1616–1682 m. See Fig. 13.

#### Biology.

This species is associated with seepage and waterfall habitats in the Andes, where it has been collected at several sites in wet rock. See Fig. 14 A–D.

### Katasophistes superficialis sp. n.

Taxon classificationAnimaliaColeopteraHydrophilidae

http://zoobank.org/D0584735-1F2E-440E-9C76-3ADC14CFDFD6

Figs 10E–H ; 12E ; 13 ; 14E, F

#### Type material examined.

**Holotype (male)**: “**ECUADOR**: **Pastaza Province**: “AGIP platform Villano B/ along transect 1 & 2/ 24.v.2008; leg. A.E.Z. Short/ small forest stream AS-08-08b” (PUCE). **Paratypes (1): ECUADOR**: Same data as holotype (1, SEMC, DNA voucher SLE 1189).

#### Differential diagnosis.

*Katasophistes superficialis* can be easily differentiated by its size (~4.5 mm), elongate oval shape, the shallow rows of enlarged systematic punctures along the elytra and its relatively long and slender maxillary palps.

#### Description.

Body length 4.5 mm, width 2.4 mm. Body elongate oval, weakly convex (Fig. 10 E, F). General coloration uniformly brown. Each elytron with five rows of shallow enlarged systematic punctures. Apex of fifth abdominal ventrite weakly emarginated. Aedeagus (Fig. 12E) with basal piece nearly 1.1-times the length of parameres; parameres at widest point, nearly 0.4-times greatest width of median lobe; apex of parameres rounded; apex of median lobe forming an acute angle.

#### Etymology.

Named in reference to the shallowness of the enlarged systematic punctures along the elytra, with the Latin word *superficialis* meaning shallow.

#### Distribution.

Ecuador (Pastaza). See Fig. 13.

#### Biology.

This species was collected in forested stream pools with abundant detritus in lowland rainforest. See Fig. 14E, F.

### Nanosaphes gen. n.

Taxon classificationAnimaliaColeopteraHydrophilidae

http://zoobank.org/92C19CA3-E66E-4339-8B36-D9D4BC0A196B

**Figure 15.**
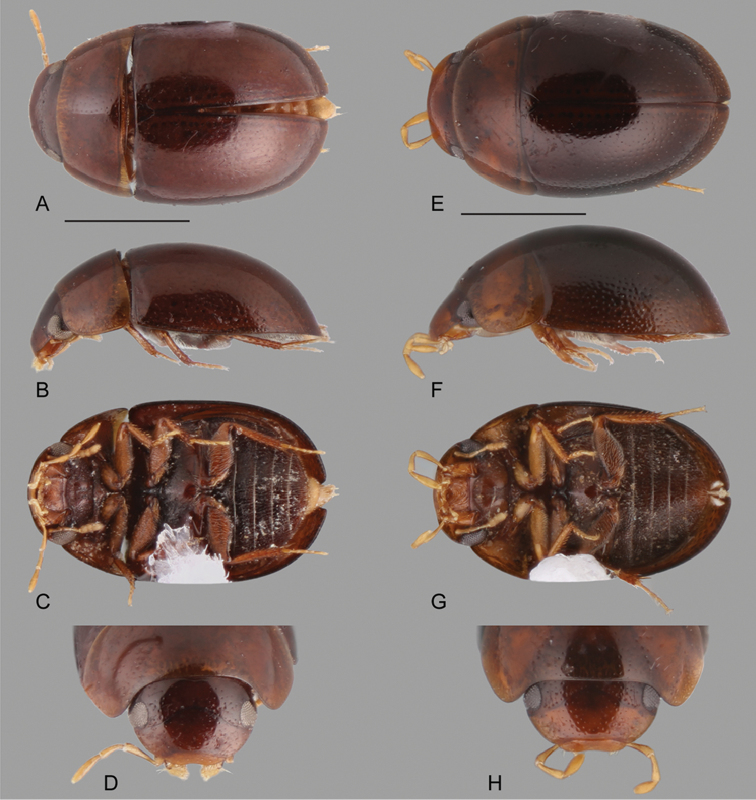
Habitus of *Nanosaphes* spp.: **A–D** *Nanosaphes castaneus*: **A** dorsal view **B** lateral view **C** ventral view **D** head **E–H** *Nanosaphes punctatus*: **E** dorsal view **F** lateral view **G** ventral view **H** head. Scale bars: 0.5 mm.

**Figure 16.**
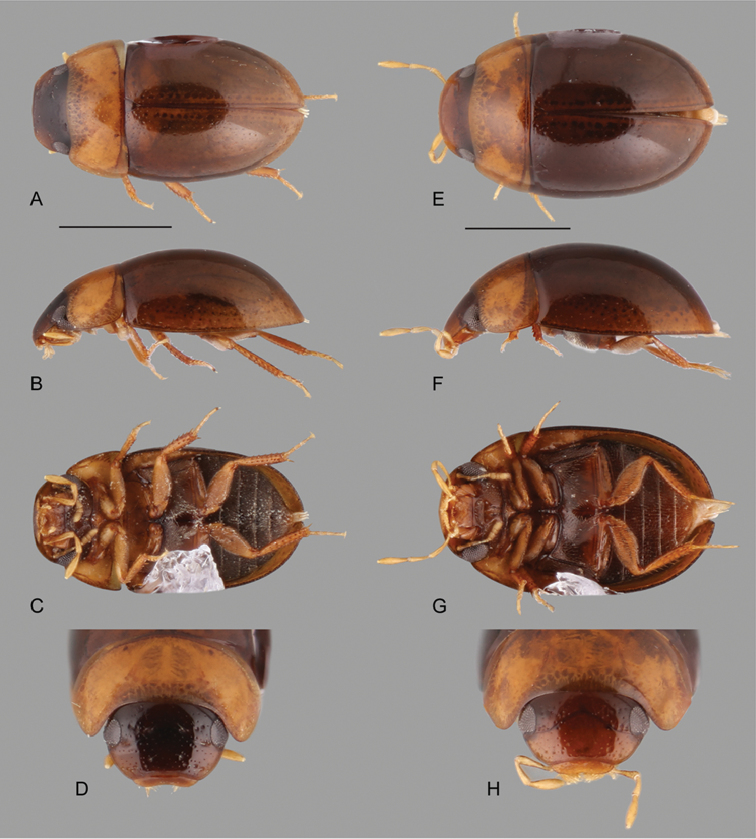
Habitus of *Nanosaphes* spp.: **A–D** *Nanosaphes tricolor*: **A** dorsal view **B** lateral view **C** ventral view **D** head **E–H** *Nanosaphes hesperus*: **E** dorsal view **F** lateral view **G** ventral view **H** head. Scale bars: 0.5 mm.

**Figure 17.**
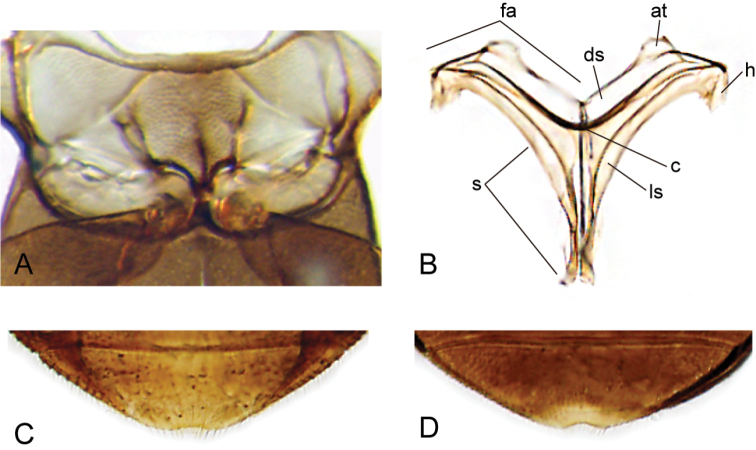
Thorax and abdomen of *Nanosaphes* spp.: **A–C** *Nanosaphes hesperus*: **A** ventral view of mesoventrite (white arrow pointing longitudinal carina) **B** posterior view of metafurca: (at) anterior tendon, (c) crux, (ds) dorsal sheath, (fa) furcal arm, (h) hemiductus, (ls) lateral sheath, (s) stalk **C** fifth abdominal ventrite **D** *Nanosaphes punctatus*, fifth abdominal ventrite.

**Figure 18.**
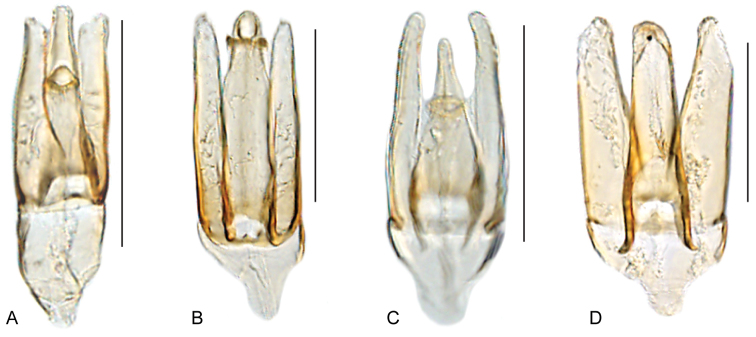
Aedeagus of *Nanosaphes* spp.: **A** *Nanosaphes tricolor* **B** *Nanosaphes hesperus* **C** *Nanosaphes castaneus* **D** *Nanosaphes punctatus*. Scale bars: 0.1 mm.

**Figure 19.**
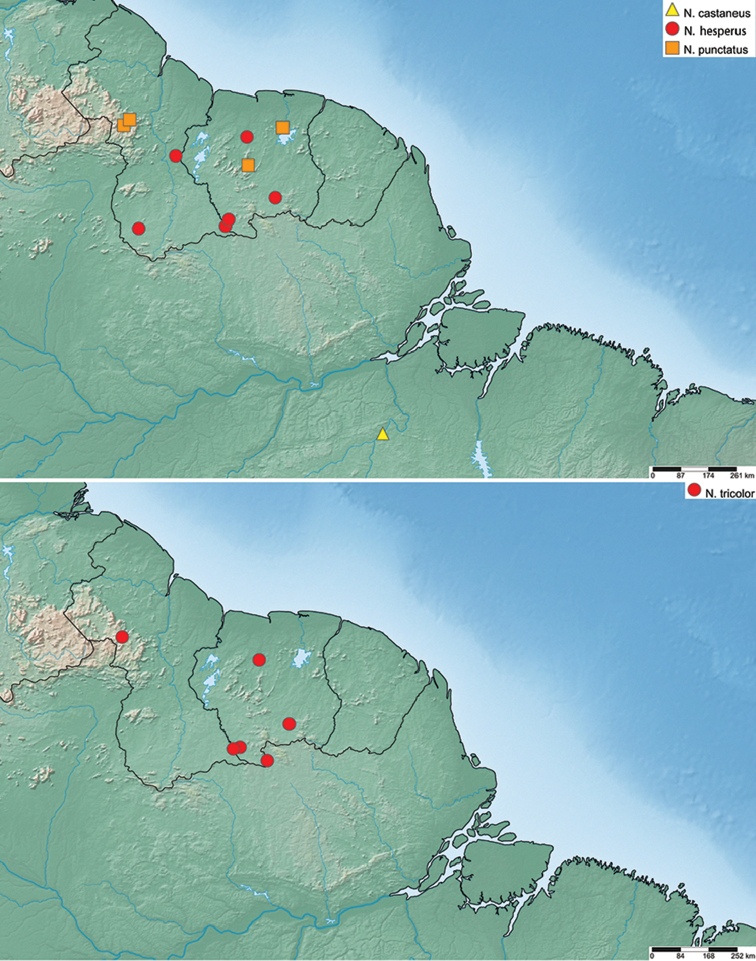
Distribution of *Nanosaphes* species.

**Figure 20.**
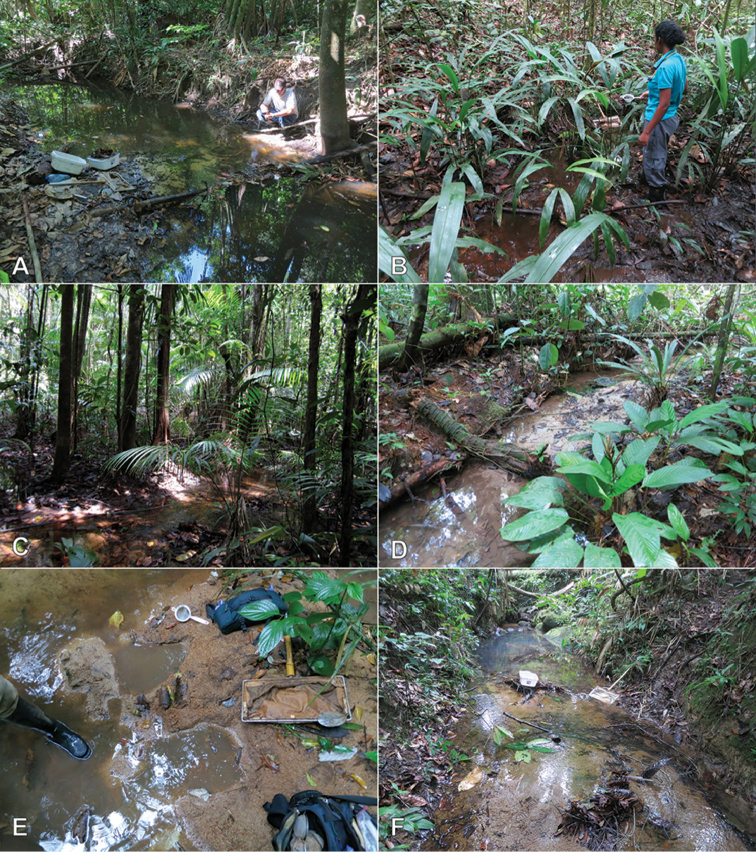
Habitat of *Nanosaphes* spp. **A** Habitat for *N. hesperus*, Guyana: near Parabara; collecting event GY13-1103-02A **B** Habitat of *N. punctatus*, Guyana, upper Potaro near Chenapau, collecting event GY14-0311-02A **C** Type locality and habitat for *N. hesperus*, Suriname, along Kutari River, collecting event SR10-0820-01A **D** Habitat for *N. hesperus* and *N. tricolor*, Suriname, near Kasikasima, collecting event SR12-0320-02A **E** Habitat for *N. tricolor*, Suriname, near Voltzberg, collecting event SR12-0729-02C **F** Habitat for *N. tricolor*, Suriname, Sipaliwini Savanna, collecting event SR17-0331-01B.

Figs 5C, D ; 15 , 16 , 17 , 18 , 19 , 20

#### Type species.

*Nanosaphes tricolor*

#### Differential diagnosis.

Very small beetles, total body length 1.15–1.45 mm, width 0.7–0.9 mm. Coloration uniformly brown, to variable along the body (see Figs 15–16). Body shape oval in dorsal view; uniformly, slightly to moderately convex in lateral view (e.g., Figs 15F, 16F). Antennae with eight antennomeres (e.g., Fig. 16G). Maxillary palps curved inward, moderately long (e.g., Fig. 16H). Each elytron with ground punctures usually only shallowly marked, seemingly forming longitudinal rows (e.g., Fig. 16E), with irregularly distributed systematic punctures bearing rather long setae, denser along lateral and posterior regions; elytra without sutural striae. Posterior elevation of mesoventrite, usually only projected as a low and short longitudinal carina between mesocoxae (Figs 5C, 17A; sharply carinated and posteriorly pointed in *N. hesperus*). Metaventrite with posterolateral and mesal glabrous patches (Fig. 5C). Posterior femora for the most part densely covered by setae. Fifth abdominal ventrite apically emarginated, with stout setae (Figs 5D, 17C–D).

The minute size of *Nanosaphes* make them smaller than any other Acidocerinae in the New World, and about equal in size as the smallest *Agraphydrus* species in the Old World. They are among the smallest water scavenger beetles worldwide. The lack of elytral serial or sutural striae and the antennae with eight antennomeres also separate *Nanosaphes* from all other Neotropical Acidocerinae genera except the co-occurring *Globulosis*. The genus can be easily separated from *Globulosis* by its smaller size and narrower, more parallel sided body form (broader and almost rotund in *Globulosis*, see Short et al. (2017)).

#### Description.

Body oval, uniformly and usually weakly convex (moderately convex in *N. punctatus*, see Fig. 15F), coloration uniformly brown, or variable along the body (e.g., paler pronotum in *N. hesperus* and *N. tricolor*, see Fig. 16). **Head.** Frons and clypeus (e.g., Fig. 16H) with shallow ground punctures irregularly distributed over the surface, accompanied by scattered seta-bearing systematic punctures; setae particularly noticeable on frons behind frontoclypeal suture; surface between punctures smooth, at most only finely reticulated along anterior and lateral margins of clypeus; anterior corners of clypeus roundly angulated; anterior margin of clypeus widely roundly emarginate. Eyes oval (e.g., Fig. 16D). Labrum rather short and wide, fully exposed and positioned nearly perpendicular to clypeus (e.g., Fig. 16B); dorsal surface convex, basally smooth, apically and laterally finely reticulated, with scattered shallow punctures; anterior margin mesally emarginate and bent inwards; anterolateral margins bearing a row of long setae. Temporae densely covered by setae (hydrofuge pubescence), with few longer setae along outer posteroventral margin of eye. Mentum nearly 1.5–1.7-times wider than long, parallel sided, basilaterally flat, with lateral oblique longitudinal ridges; anterior margin with wide, deep, concave median impression. Submentum rather flat, with sparse rather long setae; ocular ridge well-developed (e.g., Fig. 16G). Maxilla (e.g., Fig. 15G) with ventral surface of cardo and stipes smooth and shiny, with a row of stiff decumbent spiniform setae along outer dorsal margin of palpifer; limit between cardo and stipes oblique; maxillary palps yellowish, longer than antennae; palpomere 1 extending beyond base of cardo, with inner margin straight, only slightly concave at base, and outer margin distally convex; palpomere 2 similar in shape to palpomere 1, 0.7-times as long; palpomere 3 fusiform, slightly longer and wider than palpomere 1, apically widely rounded; apex of palpomere 3 bearing sensilla. Mandibles with apex bifid (examined in *N. hesperus*). Labial palps yellowish, nearly as long as mentum, dorsoventrally flattened; palpomere 2 with inner and outer margin convex apicad of midpoint; palpomere 3 digitiform, markedly shorter and narrower than palpomere 2, with a long subapical seta on outer corner. Antennae (e.g., Fig. 16G) with eight antennomeres, paler than general coloration of head; antennomere 1 anteriorly swollen near anterior margin of eye, reaching midpoint of ventral surface of eye (reaching cardo-stipes joint), nearly 1.4-times longer than antennomere 2; antennomere 2 slightly longer than antennomeres 3–4 combined; antennomere 5 forming a rather small, but well differentiated cupule; antennomeres 6–8 similar in size (7 shortest, 8 longest), slightly flattened, forming a loosely articulated, pubescent club; setae at apex of antennomere 8 longer than general pubescence of club. **Thorax.** Pronotum widest at base, narrowed anteriorly, surface evenly convex; ground punctation shallow, uniformly sparse, with surface between punctures smooth and shiny; seta bearing systematic punctures particularly noticeable as transverse anterolateral and mediolateral bands. Scutellar shield of moderate size, triangular, nearly as long as wide, with scarce shallow punctures. Prosternum (e.g., Fig. 16G) very short, flat, at most only weakly convex, not carinate; anterior margin of prosternum mesally projected, with a preapical fringe of setae; intercoxal process truncate, aligned with posterior margin of procoxal cavities. Mesoventrite not fused to mesepisterna, with anterior margin nearly as wide as anterior margin of mesepisternum; posterior elevation of mesoventrite either flat (as in *N. punctatus*), or longitudinally carinate (weakly as in *N. tricolor* (Figs 5C, 17A) and *N. castaneus* or sharply as in *N. hesperus*); mesepisternum obliquely widely concave; surface of mesoventrite and mesepisternum reticulated; mesepimeron narrow and trapezoid, with pubescent surface. Mesofurca (examined in *N. hesperus*) with short arms, 1.3-times longer than length of mesocoxae; each arm expanding ventrally as a lamina, reaching mesoventrite; apex of arms free (not forming part of lamina), oval and explanate. Metaventrite (Fig. 5C) posteromesally elevated, with elevation rather flat and metathoracic discrimen well defined; anterior and lateromedian surfaces of metaventrite pubescent, with median and posterolateral glabrous patches. Metepisterna 3–4-times longer than wide, slightly narrowing posteriorly. Metepimeron triangular and acute. Metafurca (Fig. 17B, examined in *N. hesperus*) 1.25-times wider than long, with furcal arms (fa) nearly as long as stalk (s); stalk triangular (wider near the crux (c), gradually narrowing ventrally), with paired longitudinal keels extending along basal two thirds of posterior face, and a well-developed median keel on the anterior face extending to anterior margin of dorsal sheets (ds); outer margins of stalk diverging from ventral third towards midpoint of furcal arms; furcal arms somewhat rectangular, with apex (hemiductus (h)) slightly explanate, perpendicularly positioned; anterior tendons (at) inserted along half third of dorsal edge of furcal arms; dorsal sheaths well-developed, slightly wider than widest point of lateral sheaths (ls). **Elytra.** Surface even (without elevations or depressions), with serial punctures seemingly longitudinally aligned, usually shallowly marked (e.g., Fig. 16E) (except in *N. punctatus* which has rather coarsely punctate elytra, see Fig. 15 E, F), not impressed into striae; seta bearing systematic punctures scattered along interstriae; setae of systematic punctures rather long; elytral margins simple (as opposed to explanate). Epipleura well-developed, nearly glabrous, at most with scarce setae, oblique, anteriorly wide, gradually narrowing posteriorly, extending up to line of first abdominal ventrite; pseudepipleura reduced, limited to margin of elytra. Hind wings well-developed. **Legs.** All femora with dense pubescence, along basal three fourths, remainder of surface glabrous and shiny; all femora antero-posteriorly flattened; metafemora with rather sharp tibial grooves along apical half. Tibiae moderately slender, rather weakly flattened, with moderately fine and sparse spines. All tarsi with five tarsomeres, bearing few long apical hair-like setae on dorsal face, and two lateral rows of spines on ventral face of tarsomeres 2–4; tarsomeres 1–4 similar in size and shape; tarsomere 5 approximately as long as tarsomeres 3–4 combined, without spines on ventral face; claws rather large, curved; empodium well-developed, bearing a pair of long, curved apical setae. **Abdomen.** Abdomen with five ventrites, medially longitudinally weakly convex, all ventrites with uniform, fine pubescence, either dense (as in *N. tricolor*, see Fig. 16C) or scanty (as in *N. castaneus*, *N. hesperus* and *N. punctatus*; e.g., Fig. 16G); posterior margin of fifth ventrite mesally weakly emarginated, set with a row of thick, flat spine-like setae (Figs 5D, 17C, D). Aedeagus (Fig. 18) nearly parallel sided, with basal piece between 0.3 and 0.6-times the length of parameres; median lobe with well-developed lateral basal apodemes, wider at base than base of each paramere, usually narrower at apex than preapical width of parameres; apex of median lobe rounded; parameres from slightly shorter to longer than median lobe, and only narrowing at apex; gonopore situated beyond midpoint of median lobe.

#### Larvae.

The immature stages are unknown.

#### Etymology.

Noun in apposition. Named after the small size of the beetles (the smallest known acidocerines), with the Greek word *nanos* meaning little and the Greek word *saphes* meaning distinct, in reference to the relative ease of recognizing the species of the genus. Genus is to be treated as neutral.

#### Distribution.

Brazil (Pará), Guyana, Suriname. See Fig. 19.

#### Biology.

Species are associated with stream margins, particularly where there are banks for margins of sand and roots.

#### Characters of taxonomic importance for *Nanosaphes*.

In contrast to some to of the other acidocerines treated here, the known species of *Nanosaphes* are diagnosable by external characters alone.

***Punctation*.** The well-marked ground and serial elytral punctures differentiate *N. punctatus* from the remaining species, in which the punctation is only shallowly marked.

***Coloration*.** The most common and widespread species of *Nanosaphes* (*N. tricolor* and *N. hesperus*) have a distinctive yellow coloration on the pronotum, and can be distinguished from each other by the coloration of the head. The entire body of *N. castaneus* is uniform in coloration.

***Posterior elevation of mesoventrite*.** In *Nanosaphes* the overall shape and sharpness of the carina formed in the posterior elevation of the mesoventrite aids species identification, where only *N. hesperus* has a sharp carina.

***Density of abdominal pubescence*.** It is uncommon in the Acidocerinae that this character varies. Only *Nanosaphes tricolor* has densely pubescent abdominal ventrites, whereas the remainder species of the genus the pubescence is sparser.

#### Key to the species of *Nanosaphes*

**Table:** 

1	Elytra with well-marked punctures (Fig. 15E, F); posterior elevation of mesoventrite flat	***N. punctatus***
–	Elytra with shallowly marked punctures (e.g., Fig. 15A, B); posterior elevation of mesoventrite weakly to sharply carinate	**2**
2	Coloration of the body rather uniformly brown (Fig. 15A–D)	***N. castaneus***
–	Coloration of pronotum yellow (see Fig. 16)	**3**
3	Coloration of the head and clypeus uniformly dark brown (Fig. 16D); posterior elevation of mesoventrite weakly carinate; ventral surface densely pubescent (Fig. 16C)	***N. tricolor***
–	Coloration of the head brown, with orange clypeus (Fig. 16H); posterior elevation of mesoventrite sharply carinate; ventral surface scantily pubescent (Fig. 16G)	***N. hesperus***

### Nanosaphes castaneus sp. n.

Taxon classificationAnimaliaColeopteraHydrophilidae

http://zoobank.org/7B7969CA-49EB-495B-AEF2-4BF1BC8A74C9

Figs 15A–D ; 18D ; 19

#### Type material examined.

**Holotype (male)**: “**BRAZIL: Pará**: Rio Xingu/ Camp (52°22'W, 3°39'S)/ Altamira (ca 60km S.)/ 12 Oct 1986/ P. Spangler & O. Flint// Colln. #21, pond at 2^nd^/ palm grove on trail 1” (USNM). **Paratypes (3): BRAZIL: Pará**: Rio Xingu, Camp (52°22'W, 3°39'S)/ Altamira (ca 60km S.)/ 12 Oct 1986/ P. Spangler & O. Flint (1, USNM); same data as holotype (2, USNM, SEMC).

#### Differential diagnosis.

*Nanosaphes castaneus* can be easily recognized by its smooth elytra (as opposed to rather coarsely punctate as in *N. punctatus*, compare Fig. 15A, B to Fig. 15E, F), and the uniform brown coloration along the body (as opposed to having yellow pronotum and brown elytra (as in *N. hesperus* and *N. tricolor*, compare Fig. 15A–D to Fig. 16).

#### Description.

Body length 1.3–1.4 mm, width 0.8–0.9 mm. Body elongate oval, weakly convex, uniformly brown throughout (Fig. 15A, B). Dorsal surface shallowly punctate. Posterior elevation of mesoventrite weakly carinate. Pubescence of ventral surface scanty. Aedeagus (Fig. 18C) with basal piece 0.4-times the length of parameres; parameres longer than median lobe, with rounded apex; gonopore situated near apical third of median lobe.

#### Etymology.

Named in reference to the uniform brown coloration along the body, with the Latin word *castaneus* meaning brown, of the color of chestnuts.

#### Distribution.

Brazil (Pará). Only known from one locality. See Fig. 19.

#### Biology.

In referencing Spangler’s original field notes, these specimens were collected in forested pond.

### Nanosaphes hesperus sp. n.

Taxon classificationAnimaliaColeopteraHydrophilidae

http://zoobank.org/30B9FA31-A93F-4004-A94B-187EB6AD1070

Figs 16E–H ; 17A–C ; 18B ; 19 ; 20A, C, D

#### Type material examined.

**Holotype (male)**: “**SURINAME**: Sipaliwini District/ 2°10.521'N, 56°47.244'W, 228 m/ Camp 1, on Kutari River; leg. Short/ & Kadosoe; forest stream/ 20.viii.2010; SR10-0820-01A/ 2010 CI-RAP Survey” (NZCS). **Paratypes (38): GUYANA: Region IX**: “2°06.492'N, 59°13.653'W, 274 m/ Parabara, N. side of river/ small flowing forested creek/ detritus margins & leaf packs/ leg. Short, Isaacs, Salisbury/ 3.xi.2013; GY13-1103-02A” (1, SEMC); **Region VI**: “4°09.143'N, 58°11.207'W, 105 m/ Upper Berbice, c. 1 km W/ Basecamp 1; washing sand/ banks; leg. A. Short; 22.iv.2014/ GY14-0921-03E” (1, SEMC); “margins of creek/ leg. Short, Salisbury, La Cruz/ 22.iv.2014/ GY14-0921-03H” (1, SEMC). **SURINAME: Sipaliwini District**: “2°21.776'N, 56°41.861'W, 237 m/ Camp 3, Wehepai; leg. Short &/ Kadosoe; sandy forest creek/ 4–6.ix.2010; SR10-0904-01A/ 2010 CI-RAP Survey” (19, SEMC); same, except “small stream/ 5.ix.2010; SR10-0905-01A” (1, SEMC); same, except “2°10.521'N, 56°47.244'W, 228 m/ Camp 1, on Kutari River/ Short & Kadosoe; forest stream/ 19.viii.2010; SR10-0819-02A” (1, SEMC); same, except “20.viii.2010; SR10-0820-01A” (8, SEMC); same, except “2.97731°N, 55.38500°W, 200 m/ Camp 4 (low), Kasikasima; sandy/ stream on trail to METS camp/ 20.iii.2012; SR12-0320-02A/ leg. A. Short; 2012 CI-RAP Survey” (3, SEMC, including DNA voucher SLE485); “04°40.966'N, 56°11.262'W, 96 m/ Raleighfallen Nature Reserve/ plateau below Voltzberg; rock pool/ leg. A. Short, Maier & McIntosh/ 28.vii.2012; SR12-0728-01F” (1, SEMC); “04°40.910'N, 56°11.138'W, 78 m/ Raleighfallen Nature Reserve/ Voltzberg trail; margin of stream/ leg. C. Maier & V. Kadosoe/ 30.vii.2012; SR12-0730-01A” (1, SEMC); Raleighfallen Nature Reserve, 17.iii.2016, leg. Girón, SR16-0317-04A (1, SEMC, DNA voucher SLE1070).

#### Differential diagnosis.

*Nanosaphes hesperus* (Fig. 16E–H) can be easily recognized by its smooth elytra (as opposed to rather coarsely punctate as in *N. punctatus*, see Fig. 15E, F), and the coloration pattern along the body, with brown elytra, yellow pronotum and brown head with orange clypeus (as opposed to uniform brown coloration along the body as in *N. castaneus*, or uniformly dark brown head (including the clypeus) as in *N. tricolor*, see Fig. 16D). Furthermore, it differs from the remaining known species by the sharp, strongly elevated, and pointed carina on the mesoventrite (as opposed to weakly elevated).

#### Description.

Body length 1.3–1.5 mm, width 0.7–0.9 mm. Body elongate oval, weakly convex, with brown head, orange clypeus, yellow pronotum and brown elytra (Fig. 16E–H). Dorsal surface shallowly punctate. Posterior elevation of mesoventrite sharply carinate. Pubescence of ventral surface scanty. Aedeagus (Fig. 18B) with basal piece 0.35-times the length of parameres; parameres as long as median lobe, with narrowly round apex; gonopore situated near apex of median lobe.

#### Etymology.

Named in reference to the gradation of orange to brown colorations along the body of the beetles, which resembles the colors of the sunset, with the Latin word *hesperus* meaning evening.

#### Distribution.

Guyana, Suriname. Elevation range 105–228 m. See Fig. 19.

#### Biology.

This species has been collected along the margins of forested streams (see Fig. 20A, C, D.

### Nanosaphes punctatus sp. n.

Taxon classificationAnimaliaColeopteraHydrophilidae

http://zoobank.org/59E30D06-ADB4-4C39-8AE4-4ED03ED8F58F

Figs 15E–H ; 17D ; 18D ; 19 ; 20B

#### Type material examined.

**Holotype (male)**: “**SURINAME**: Sipaliwini District/ 04°56.871'N, 55°10.911'W, 462 m/ Brownsberg Nature Park, forested/ stream with lots of detritus; leg./ Short, Maier, McIntosh; 4.viii.2012/ SR12-0804-01A” (NZCS). **Paratypes: (40): GUYANA: Region XII**: “5°0.730'N, 59°38.965'W, 585 m/ Upper Potaro Camp I (c. 7 km/ NW Chenapau), Ridge Trail/ leg. Short, Baca, Salisbury/ 11.iii.2014/ GY14-0311-02A” (32, SEMC, CBDG); “5°10.514'N, 59°28.970'W, 440 m/ Kaieteur National Park, trail by guest-/ house; forest pools; leg. Short/ Salisbury, La Cruz; 21.iii.2014/ GY14-0321-01B” (1, SEMC). **SURINAME: Sipaliwini District**: same data as holotype (6, SEMC, including DNA vouchers SLE 507 and 508); “3°55'36.0012”, -56°11'17.9952”, 600 m/ Central Suriname Nature Reserve: Tafelberg Summit, nr. Augustus Creek Camp; 17.viii.2013; leg. Short & Bloom; SR13-0817-01A” (1, SEMC, DNA voucher SLE 1069).

#### Differential diagnosis.

*Nanosaphes punctatus* is readily recognized among its congeners by its coarse elytral ground punctation (as opposed to shallowly punctate) (see Fig. 15E, F). In addition, the dorsal outline in lateral view is more convex in comparison.

#### Description.

Body length 1.2–1.4 mm, width 0.7–0.9 mm. Body elongate oval, moderately convex, uniformly brown throughout (see Fig. 15E–H). Dorsal surface coarsely punctate. Posterior elevation of mesoventrite flat. Pubescence of ventral surface scanty. Aedeagus (Fig. 18D) with basal piece 0.4-times the length of parameres; parameres nearly as long as median lobe, with round apex; gonopore situated near apex of median lobe.

#### Etymology.

Named in reference to the relative coarseness of the dorsal punctation of the species, with the Latin word *puncta* meaning puncture.

#### Distribution.

Guyana, Suriname. Elevation range 440–585 m. See Fig. 19.

#### Biology.

This species was collected in small forested streams and pools with abundant detritus (Fig. 20B).

### Nanosaphes tricolor sp. n.

Taxon classificationAnimaliaColeopteraHydrophilidae

http://zoobank.org/8A659E25-1129-4A7E-A696-3FD01BC7E3B6

Figs 5C, D ; 16A–D ; 18A ; 19 ; 20D–F

#### Type material examined.

**Holotype (male)**: “**SURINAME**: Sipaliwini District/ 2.97731°N, 55.38500°W, 200 m/ Camp 4 (low), Kasikasima; sandy/ creek, trail to Kasikasima; leg. A. Short/ 22.iii.2012; SR12-0322-02A/ flotation; 2012 CI-RAP Survey” (NZCS). **Paratypes (136): GUYANA: Region XII**: “5°17.823'N, 59°50.000'W, 684 m/ Ayanganna Airstrip, trail from/ Blackwater Creek Camp to Potaro/ River; sand/gravel washing in/ gravel bar; leg. A. Short / 18.iii.2014; GY14-0318-03C” (1, SEMC; DNA voucher SLE1067). **SURINAME: Sipaliwini District**: “2°21.776'N, 56°41.861'W, 237 m/ Camp 3, Wehepai; leg. Short &/ Kadosoe; sandy forest creek/ 4–6.ix.2010; SR10-0904-01A/ 2010 CI-RAP Survey” (47, SEMC, including DNA voucher SLE130); same, except “small stream/ 5.ix.2010; SR10-0905-01A” (1, SEMC); same, except “2°19.280'N, 56°52.595'W, 224 m/ Rapids on Kutari River/ leg. A.E.Z. Short; forest stream/ 18.viii.2010; SR10-0818-01A” (1, SEMC); same, except “2.97731°N, 55.38500°W, 200 m/ Camp 4 (low), Kasikasima; sandy/ stream on trail to METS camp/ 20.iii.2012; SR12-0320-02A” (4, SEMC); same data as holotype (9, SEMC); same, except “04°42.480'N 56°13.159'W, 24 m/ Raleighfallen Nature Reserve, trail/ to Raleighfallen; creek margins/ leg. Short, McIntosh, & Kadosoe/ 27.vii.2012; SR12-0727-03A” (3, SEMC); same, except “04°40.910'N, 56°11.138'W, 78 m/ Raleighfallen Nature Reserve / Voltzberg Station; sand bar in/ stream; leg. C. McIntosh / 29.vii.2012; SR12-0729-02C” (8, SEMC); same, except “Votlzberg trail; margin of stream; leg. C. Maier, V. Kadosoe; 30.vii.2012; SR12-0730-01A” (1, SEMC); “2°00.342'N, 55°58.149'W, 337 m/ Sipaliwini Savanna Nature Res./ 4-Brothers Mts.; clearwater stream/ sandy w/detritus; 31.iii.2017/ leg. Short & Baca; SR17-0331-01B” (32, SEMC); same, except “sandy w/ emergent veg; SR17-0331-01C” (19, SEMC); same, except “at night; leg. Short; SR17-0331-01F” (10, SEMC).

#### Differential diagnosis.

*Nanosaphes tricolor* can be easily recognized by its smooth elytra (as opposed to rather coarsely punctate as in *N. punctatus*, see Fig. 15E, F), and the coloration pattern along the body, with uniformly dark brown head (including the clypeus, see Fig. 16D), yellow pronotum and brown elytra (see Fig. 16A, B; as opposed to uniform brown coloration along the body as in *N. castaneus*, Fig. 15E, H). It is similar to *N. hesperus* in the paler coloration of the pronotum, but differs from it by the uniform and darker coloration of the head (orange in *N. hesperus*) and the weak development of the longitudinal elevation of the mesoventrite (as opposed to strongly elevated as to form a sharp and pointed carina in *N. hesperus*).

#### Description.

Body length 1.1–1.4 mm, width 0.7–0.75 mm. Body elongate oval, weakly convex, with uniformly dark brown head (including clypeus, Fig. 16D), yellow pronotum and brown elytra (Fig. 16A, B). Dorsal surface shallowly punctate. Posterior elevation of mesoventrite weakly carinate. Pubescence of ventral surface very dense. Aedeagus (Fig. 18A) with basal piece nearly 0.6-times the length of parameres; parameres slightly shorter than median lobe, with obliquely round apex; gonopore situated near apical third of median lobe.

#### Etymology.

Noun in apposition. Named after the three colors present along the body of the beetles (uniformly dark head, yellow pronotum and brown elytra), with the Latin prefix *tri* meaning three and the Latin word *color*.

#### Distribution.

Guyana, Suriname. Elevation range 24–684 m. See Fig. 19.

#### Biology.

This species has been collected along the margins of forested streams (see Fig. 20D, F).

### Chasmogenus

Taxon classificationAnimaliaColeopteraHydrophilidae

Sharp, 1882

Chasmogenus Sharp, 1882: 72.
Dieroxenus Spangler, 1979: 753. **Syn. n.**

#### Discussion.

**Figure 21.**
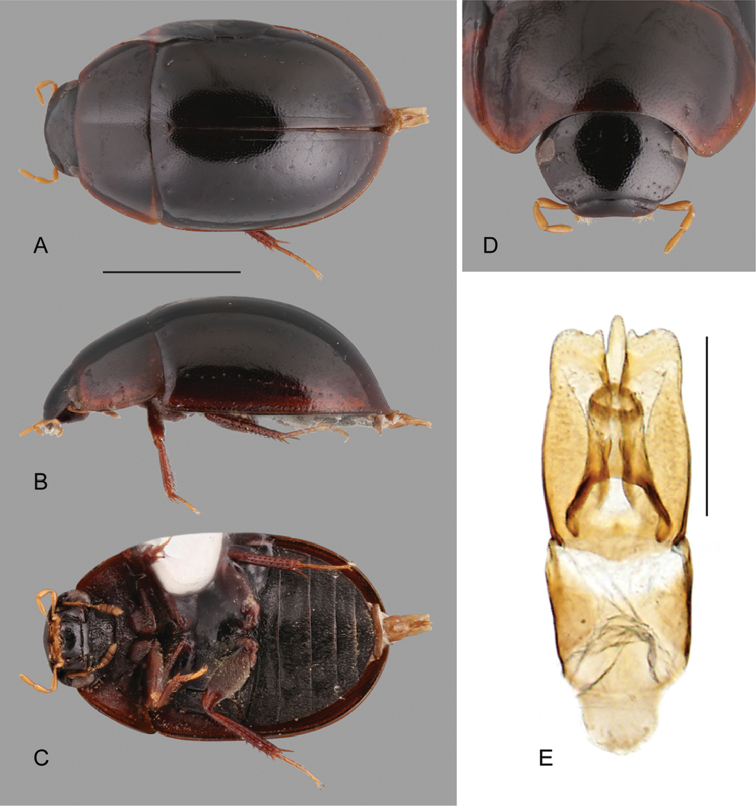
Habitus of *Chasmogenus cremnobates*: **A** dorsal view **B** lateral view **C** ventral view **D** head **E** aedeagus. Scale bar 1 mm.

In comparing the new taxa described here to existing genera, we observed striking similarities between the monotypic Andean genus *Dieroxenus* Spangler and the widespread genus *Chasmogenus* Sharp. *Dieroxenus* shares most diagnostic characters of New World *Chasmogenus*, including general body size, the presence of sutural striae (extremely rare in Acidocerinae), antennae with eight antennomeres, a longitudinal carina on the mesoventrite, and a “simple” tri-lobed aedeagus (Hebauer 1992). It does differ from *Chasmogenus* in a few respects: *Dieroxenus* has a slightly more robust body form, slightly shorter maxillary palps, and its elytral systematic punctures are enlarged and easily distinguished from the ground punctation. However, most if not all of these features could be attributed to the unusual seep-inhabiting nature of this species, while other *Chasmogenus* are known from streams and marshes. Additionally and perhaps most convincing morphologically, is that the aedeagal form of *Dieroxenus* (Fig. 21E) matches that of some Neotropical *Chasmogenus* (e.g., see Figs 1–4 in Short 2005).

When *Dieroxenus* was described nearly 40 years ago, the systematics and classification of the Hydrophilidae was radically different. *Chasmogenus* was then considered part of *Helochares*, and most genera that are now in the Acidocerinae were assigned to different tribes than they are today. Consequently, Spangler (1979) did not consider what we now call *Chasmogenus* when making his comparisons, and instead focused on its similarities and differences from *Enochrus*, which we now know is not very closely related (Short and Fikáček 2013).

### Chasmogenus cremnobates

Taxon classificationAnimaliaColeopteraHydrophilidae

(Spangler, 1979) comb. n.

Fig. 21

Dieroxenus cremnobates Spangler, 1979: 754

#### Type material examined.

**Holotype**: “ECUADOR: Napo, Baeza (72 km E), 16 May 1975, Spangler, Langley, and Cohen” (USNM). **Paratypes: ECUADOR: Napo**: same data as holotype (44 males; 42 females); **Tungurahua**: “Baños (35 km E), 29 May 1975, Langley, Cohen, and Monnig (8 males; 6 females); “Baños (18 km E), 25 Jan 1976, Spangler, et al. (15 males; 18 females); “Baños (20 km E), 28 Jan 1976, Spangler, et al. (7 males; 6 females); “Baños (39 km E), 28 Jan 1976, Spangler, et al. (2 females).

#### Additional material.

**ECUADOR: Napo**: “San Francisco de / Borja 17 Jan 1978/ 1610 m PJ Spangler/ & J. Anderson // seep on stream bank” (6 males, 6 females; USNM).

#### Key to the genera of New World Acidocerinae

**Table:** 

1	Eyes absent. Known only from a cave in Ecuador	***Troglochares***
–	Eyes present	**2**
2	Eyes completely divided into dorsal and ventral sections by a lateral projection of frons. Size small (<3 mm)	***Quadriops***
–	Eyes not divided into dorsal and ventral sections by frons. Size variable	**3**
3	Labrum concealed by clypeus, elytral margins broadly explanate. Body extremely dorsoventrally compressed	***Helobata***
–	Labrum not concealed by clypeus elytral margins not or at most weakly explanate. Body form variable but rarely dorsoventrally compressed	**4**
4	Elytra with distinctly impressed sutural striae. Only Neotropical region	***Chasmogenus***
–	Elytra without sutural striae. Both Neotropical and Nearctic	**5**
5	Prosternum with strongly elevated median carina	***Crucisternum***
–	Prosternum without median carina; at most tectiform medially	**6**
6	Metafemora entire glabrous. Size small (<3 mm)	***Tobochares***
–	Metafemora pubescent at least on basal half or anterior third. Size variable	**7**
7	Fifth ventrite entire, without apical emargination or truncation. Antennae with nine antennomeres. Maxillary palps shorter than the width of the head	***Radicitus***
–	Fifth ventrite with apical emargination or truncation. Antennae with eight or nine antennomeres. Maxillary palps variable in length	**8**
8	Elytral systematic punctures very distinct, distinctly larger than surrounding ground punctation. Antennae with nine antennomeres	***Katasophistes***
–	Elytral systematic punctures indistinct, usually blending with surrounding ground punctation. Antennae with eight or nine antennomeres	**9**
9	Antennae with nine antennomeres. Size variable, but rarely less than 4 mm. Extremely common and widespread throughout the New World	***Helochares***
–	Antennae with eight antennomeres. Rare and only known from the Guiana Shield region of South America. Size very small (< 3 mm)	**10**
10	Body form circular, rounded. Size very small (1.9-2.3 mm)	***Globulosis***
–	Body form ovoid, parallel sided. Size exceedingly small (1.1–1.5 mm)	***Nanosaphes***

## Supplementary Material

XML Treatment for Crucisternum

XML Treatment for Crucisternum escalera

XML Treatment for Crucisternum ouboteri

XML Treatment for Crucisternum queneyi

XML Treatment for Crucisternum sinuatus

XML Treatment for Crucisternum toboganensis

XML Treatment for Crucisternum vanessae

XML Treatment for Crucisternum xingu

XML Treatment for Crucisternum

XML Treatment for Katasophistes

XML Treatment for Katasophistes charynae

XML Treatment for Katasophistes cuzco

XML Treatment for Katasophistes merida

XML Treatment for Katasophistes superficialis

XML Treatment for Nanosaphes

XML Treatment for Nanosaphes castaneus

XML Treatment for Nanosaphes hesperus

XML Treatment for Nanosaphes punctatus

XML Treatment for Nanosaphes tricolor

XML Treatment for Chasmogenus

XML Treatment for Chasmogenus cremnobates
